# Mesenchymal stromal cells in age-related neurodegenerative disorders: mechanisms, translational barriers, and precision strategies

**DOI:** 10.3389/fimmu.2026.1965598

**Published:** 2026-09-28

**Authors:** Zhiyong Long, Honghao Guo, Lingyun Sun

**Affiliations:** 1 Department of Physical Medicine and Rehabilitation, The Affiliated Panyu Central Hospital of Guangzhou Medical University, Guangzhou, China; 2 Rheumatology Medical Center and Stem Cell Intervention Center, Department of Rheumatology and Immunology, Nanjing Drum Tower Hospital, The Affiliated Hospital of Nanjing University Medical School, Nanjing, China; 3 Department of Rheumatology and Immunology, Nanjing Drum Tower Hospital, Clinical College of Nanjing Medical University, Nanjing, China

**Keywords:** ageing, extracellular vesicles, mesenchymal stromal cells, neurodegeneration, neuroinflammation, precision medicine

## Abstract

Ageing remodels the neuroimmune, metabolic, and vascular environment in ways that accelerate neuronal vulnerability and constrain tissue repair. Mesenchymal stromal cells (MSCs) and MSC-derived extracellular vesicles (MSC-EVs) are therefore attractive candidates for neurodegenerative disorders because their effects extend beyond cell replacement to coordinated paracrine, immunomodulatory, trophic, and vascular regulation. This critical review evaluates the biological rationale, disease-specific evidence, translational limitations, and precision-development strategies for MSC-based interventions in Alzheimer’s disease, Parkinson’s disease, Huntington’s disease, amyotrophic lateral sclerosis, multiple system atrophy, and vascular dementia, whilst using selected rare hereditary neurodegenerative disorders as exploratory boundary cases. Current evidence supports three interconnected modes of action: reprogramming maladaptive neuroimmune responses; preserving neuronal homeostasis through trophic support, mitochondrial rescue, and proteostasis regulation; and restoring the neurovascular unit and blood-brain barrier. However, reproducible clinical efficacy remains limited by variation in tissue source, donor age, manufacturing, potency, dose, route, and outcome selection, as well as poor central nervous system exposure and inadequate patient stratification. Ageing-related changes in both the recipient microenvironment and the cellular product further complicate translation. Future development should replace empirical cell administration with mechanism-defined products, validated potency assays, quantitative biodistribution, biomarker-guided enrolment, engineered cells or vesicles, rational combinations, and trials using disease-modifying endpoints. MSC-based therapies are best positioned not as universal regenerative agents, but as mechanism-defined, biomarker-matched products—potentially engineered at the cell or EV level—that regulate biologically active and potentially reversible disease states.

## 1 Introduction

Ageing is the major shared risk context for Alzheimer’s disease (AD), Parkinson’s disease (PD), vascular dementia (VaD), and several other neurodegenerative syndromes, and it also shapes the onset, progression, and treatment response of Huntington’s disease (HD), amyotrophic lateral sclerosis (ALS), and multiple system atrophy (MSA). These disorders are characterised by progressive neuronal dysfunction, irreversible cell loss, impaired proteostasis, mitochondrial stress, chronic inflammation, vascular dysfunction, and limited intrinsic repair. Their rising prevalence creates substantial clinical, social, and economic burdens, whilst most available treatments remain symptomatic or provide only partial disease modification. This therapeutic gap has sustained interest in mesenchymal stromal/stem cells (MSCs), which can be isolated from bone marrow, adipose tissue, umbilical cord, dental tissues, and other sources and expanded for autologous or allogeneic use (1–8). Selected rare hereditary neurodegenerative disorders are considered only as boundary cases for testing the generalisability of MSC-mediated trophic and immunomodulatory effects; they are not treated as sharing the ageing biology, causal mechanisms, or evidence base of the core indications.

Early MSC research was dominated by the expectation that transplanted cells would engraft in the central nervous system (CNS), differentiate into neurons or glia, and replace lost tissue. Accumulating lineage-tracing and biodistribution data have substantially revised this view. Most administered MSCs survive only transiently, and durable neural differentiation or circuit integration is uncommon. Their biological effects are now attributed mainly to paracrine and immunomodulatory actions mediated by soluble factors, extracellular vesicles (EVs), neurotrophins, cytokines, lipids, nucleic acids, and, in selected experimental settings, mitochondrial transfer. These products may suppress neuroinflammation, support neuronal metabolism, improve vascular and blood-brain barrier function, and stimulate endogenous repair without requiring long-term cellular engraftment (9–16, 39, 40, 51).

This conceptual shift has expanded the therapeutic platform from intact MSCs to MSC-derived secretome and EV preparations. Cell-free products may reduce risks related to pulmonary entrapment, uncontrolled differentiation, and variable post-thaw viability, and they may be more amenable to storage, engineering, and repeated administration. However, their apparent simplicity is deceptive: EV isolation, identity, dose definition, cargo potency, biodistribution, and batch consistency remain insufficiently standardised. Across both cell-based and cell-free approaches, clinical evidence is still dominated by small, early-phase studies with heterogeneous products, routes, schedules, patient populations, and endpoints. Phase III success is rare, and neutral findings are less visible than positive preclinical reports (4, 6, 10, 12, 14, 17–19).

This critical Review evaluates the biological rationale, disease-specific evidence, translational limitations, and precision-development strategies for MSC-based interventions in neurodegenerative disorders. Rather than treating MSCs as universal regenerative agents, we position them as context-dependent regulators of the neuroimmune, metabolic, and neurovascular microenvironment. Particular attention is given to ageing biology, product heterogeneity, CNS delivery, biomarker-guided patient selection, clinically meaningful endpoints, and the transition from empirical cell administration to mechanism-defined, quantifiable therapeutic products (3, 5, 18, 20).

A central premise of this analysis is that the translational value of MSCs cannot be inferred from the breadth of their reported effects. Pleiotropy is advantageous only when it is controlled, measurable, and matched to a disease mechanism that remains biologically modifiable. The same product may be beneficial in an inflammation-dominant early phenotype, ineffective in a late stage defined by irreversible neuronal loss, and potentially counterproductive in a microenvironment that redirects its secretory programme. Accordingly, we distinguish biological plausibility from therapeutic validation and assess evidence along a hierarchy that moves from mechanistic causality and reproducible preclinical efficacy to target engagement, controlled clinical benefit, and manufacturing consistency. This framework is intended to prevent positive findings in highly selected models from being interpreted as proof of a class-wide regenerative effect (3, 5, 6, 17–20).

The review also differentiates between narrative completeness and evidentiary weight. A large number of mechanistic publications does not compensate for weak experimental design, and repeated demonstration of a favourable phenotype in closely related models does not constitute independent validation. Greater weight should be given to blinded and adequately powered studies, replication across laboratories and model systems, clinically relevant timing, comparison with standard therapy, and concordance between molecular, structural, and functional outcomes. For clinical studies, randomisation, allocation concealment, masking, complete reporting, prespecified endpoints, and product comparability are more informative than the novelty of the cell source or engineering strategy. This approach inevitably produces a more restrained interpretation than a conventional catalogue review, but it better identifies which findings are sufficiently mature to guide translational investment (5, 6, 17–20).

## 2 Biological evolution and product identity

The modern MSC concept emerged from studies of adherent bone marrow stromal populations capable of supporting haematopoiesis and forming mesenchymal tissues. Subsequent work established clonogenic culture methods, demonstrated multilineage differentiation, and broadened the range of tissue sources. The term “mesenchymal stem cell” encouraged a regenerative narrative centred on stemness and structural replacement; however, cells meeting conventional criteria are not a uniform stem-cell population. The International Society for Cellular Therapy criteria - plastic adherence, a characteristic surface-marker profile, and osteogenic, chondrogenic, and adipogenic differentiation *in vitro* - provide a practical identity framework but do not measure therapeutic potency or distinguish functionally divergent subpopulations (21–30). The subsequent ISCT position statement clarified that the acronym MSC may be retained, but the preferred term is ‘mesenchymal stromal cell’ unless stemness is demonstrated by rigorous *in vitro* and *in vivo* evidence; it also recommends explicit specification of tissue source and functional assays linked to the intended mechanism of action. A later ICCBBA–ISCT consensus further standardised tissue-source nomenclature for culture-adapted MSC products (23, 334, 335).

The historical nomenclature remains more than a semantic issue. The label ‘stem cell’ encouraged investigators to interpret morphological neural differentiation and expression of lineage-associated markers as evidence of functional neuronal conversion. Subsequent electrophysiological, lineage-tracing, and transplantation studies showed that many induced neuron-like phenotypes reflected cytoskeletal contraction, non-specific marker expression, cell fusion, or uptake of cellular debris rather than acquisition of mature action potentials, synaptic connectivity, and circuit-level function. This correction shifted the central scientific question from whether MSCs can become neural tissue to how a transient cellular product alters host biology. It also established a stricter evidentiary threshold for claims of regeneration: structural appearance is insufficient without durable lineage identity, physiological integration, and demonstrable contribution to function (21–32).

A related misconception concerns immune privilege. Low basal expression of major histocompatibility complex class II and co-stimulatory molecules reduces immediate recognition but does not render allogeneic MSCs invisible to the host. Exposure to interferon-gamma and other inflammatory signals can alter antigen presentation, whilst repeated administration can generate humoral or cellular responses and accelerate clearance. At the same time, inflammatory licensing is often required for full immunosuppressive potency. Thus, the inflammatory environment has a dual role: it activates therapeutic function and can increase immunogenicity or exhaust the product. This tension argues against universal assumptions about repeat dosing and supports direct measurement of anti-donor responses, complement activation, and product persistence in clinical programmes (31–43).

MSC heterogeneity is intrinsic and operates at several levels. Tissue origin shapes proliferative capacity, secretory composition, immunological activity, and susceptibility to senescence. Bone marrow-derived MSCs have the longest clinical history but may be affected by donor age and invasive harvesting. Adipose-derived cells are abundant and accessible but can reflect metabolic comorbidity. Perinatal-tissue MSCs often show robust expansion and low baseline immunogenicity, making them attractive for allogeneic manufacturing, whereas dental and neural crest-derived populations may display specialised neurotrophic profiles. Within each source, donor genetics, age, sex, disease, medications, lifestyle, culture medium, oxygen tension, passage number, confluence, cryopreservation, and thawing alter phenotype and function. Even a single manufacturing batch contains clones with different proliferative, secretory, and differentiation capacities (44–50).

The practical consequence of this heterogeneity is that tissue source should not be treated as a categorical proxy for potency. Umbilical cord-derived cells may, on average, expand more rapidly than adult bone marrow cells, whereas adipose-derived populations may be obtained at higher yield; neither feature guarantees superiority for a neurological indication. Donor age, inflammatory exposure, metabolic disease, collection procedure, serum or xeno-free medium, oxygen tension, passage, and cryopreservation can produce within-source differences larger than between-source averages. Importantly, no head-to-head randomised trial in a neurological indication has demonstrated superiority of one tissue source over another; current comparisons are predominantly indirect and are frequently confounded by non-identical manufacturing. A rigorous development programme should therefore compare candidate sources under a common process and assay panel, then select the product according to functional performance, scalability, and clinical feasibility rather than historical familiarity or presumed tissue-specific advantages (44–50).

These observations undermine the assumption that any product labelled “MSC” is therapeutically equivalent. Phenotypic release criteria may confirm identity and purity but cannot predict anti-inflammatory, neurotrophic, angiogenic, or metabolic activity. Functional potency assays therefore need to be linked to the intended mechanism and indication. A product developed for an inflammation-dominant phenotype may require assays of cytokine suppression, microglial modulation, or T-cell regulation, whereas a product intended for neurovascular repair may require measures of endothelial protection, barrier integrity, or angiogenic signalling. Product definition must consequently incorporate source, donor characteristics, manufacturing process, passage, viability, secretory activity, and a disease-relevant potency profile rather than relying on surface markers alone (33, 44, 45, 49–55).

The *in vivo* fate of MSCs also changed the therapeutic paradigm. After intravenous infusion, many cells are retained in the pulmonary microcirculation and are cleared within hours to days; only a small proportion reaches distal tissues, and persistent CNS engraftment is limited. Nevertheless, biological effects can outlast cell survival. Apoptotic or stressed MSCs may be phagocytosed by host immune cells, triggering secondary immunoregulatory responses, whilst viable cells release soluble mediators and vesicles during their brief residence. MSCs are therefore better described as injury-responsive signalling cells or transient biological drug factories than as durable tissue-replacement units. Their low expression of co-stimulatory molecules permits relative immune evasion, but they are not absolutely immune privileged; inflammatory licensing can alter major histocompatibility complex expression, and repeated allogeneic administration may elicit immune recognition (33, 51–55).

Transient persistence also alters the interpretation of dose. Cell number alone is a poor pharmacological metric when viability, activation state, surface procoagulant activity, secretory output, and host clearance vary substantially. Two nominally identical doses may deliver different quantities and durations of active mediators. Moreover, pulmonary sequestration after intravenous infusion is not simply a delivery failure: interactions with lung endothelium, complement, platelets, monocytes, and macrophages may initiate systemic immunological effects that subsequently influence the CNS. This ‘hit-and-run’ model is plausible, but it should not become an unfalsifiable explanation for efficacy in the absence of brain exposure. Studies must quantify product fate, circulating mediators, host-cell responses, and temporal pharmacodynamics to determine whether peripheral reprogramming, direct CNS delivery, or both are required (33, 51–55).

The clinical translation of MSCs outside neurology provides complementary lessons. Early studies in haematological and inflammatory diseases established feasibility and short-term safety, whilst later product development exposed the consequences of batch variability, loss of potency after cryopreservation, poorly defined dose-response relationships, and inadequate patient stratification. Approvals in selected jurisdictions demonstrated regulatory feasibility, but failures of confirmatory trials and market withdrawals showed that manufacturing changes and inconsistent clinical protocols can erase apparent efficacy. The first United States approval of an MSC product for paediatric steroid-refractory acute graft-versus-host disease was an important milestone, but it did not resolve the broader problems of mechanism, potency, and reproducibility. For chronic neurological disease, these lessons are particularly important because treatment effects may be modest, follow-up is prolonged, and small variations in product performance can be obscured by heterogeneous disease trajectories (56–69). The evolution from stromal support to multipotent-cell interpretation and finally to paracrine and immunometabolic regulation is summarised in **Figure 1**.

**Figure 1:**
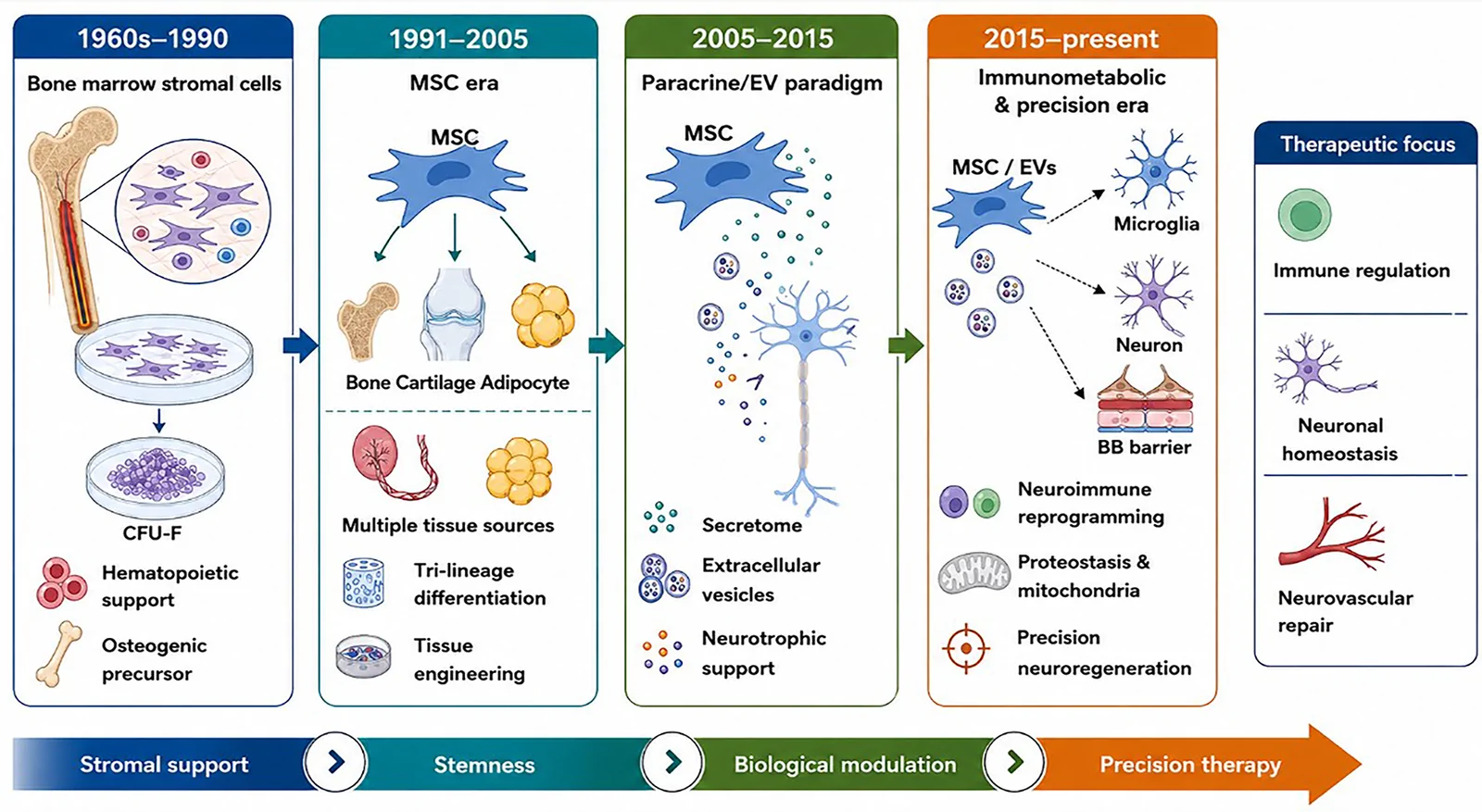
Paradigm evolution of mesenchymal stromal cell (MSC) therapy in neurodegenerative diseases. This schematic illustrates the progressive conceptual evolution of MSC-based therapies for neurodegenerative disorders from stromal support to precision regenerative medicine. In the 1960s–1990s, bone marrow-derived stromal cells were primarily recognised for haematopoietic support and osteogenic precursor activity within the bone marrow niche. From 1991–2005, the MSC era emerged, highlighting their tri-lineage differentiation potential (bone, cartilage, adipocyte) and applications in tissue engineering across multiple tissue sources. Between 2005–2015, the paracrine/extracellular vesicle (EV) paradigm became dominant, emphasising MSC-derived secretome, EVs, and neurotrophic support as key mediators of therapeutic effects. From 2015 to the present, the field has shifted towards an immunometabolic and precision-focused paradigm, in which MSCs and MSC-EVs regulate neuroimmune responses, proteostasis, mitochondrial function, neuronal homeostasis, and neurovascular unit integrity through multi-target mechanisms. The overall trajectory reflects a transition from stromal support and stemness towards biological modulation and ultimately precision neuroregenerative therapy. MSC, mesenchymal stem cell; EV, extracellular vesicle; CFU-F, colony-forming unit-fibroblast; CNS, central nervous system.

The regulatory history of non-neurological MSC products further demonstrates that approval and durable clinical value are not synonymous. Conditional or region-specific approvals may be based on severe unmet need, small populations, or surrogate evidence, whereas subsequent confirmatory studies can expose instability in potency or administration. Conversely, a negative pivotal trial does not prove that every MSC product is inactive; it may indicate that the tested process, dose, population, or endpoint was inadequate. The appropriate unit of interpretation is the specific manufactured product in a defined indication. Neurological development should therefore avoid borrowing credibility from approvals in unrelated diseases and should instead apply the lessons of those programmes to comparability, release testing, and confirmatory evidence (56–60, 62–65, 69).

## 3 Mechanisms relevant to neurodegeneration

MSC-based therapies act through overlapping neuroimmune, neurotrophic, metabolic, and vascular pathways rather than a single universal mechanism. The relative contribution of each pathway depends on cell source, manufacturing, route, dose, disease stage, and host inflammatory state. This pleiotropy is attractive in multifactorial neurodegeneration, but it also complicates causal attribution and potency testing. A clinically useful mechanism must therefore be defined at the product-indication level rather than inferred from generic MSC biology.

Mechanistic evidence should be interpreted with attention to scale. Changes in a single cytokine, microRNA, or signalling protein are frequently presented as the mechanism of action even though MSC products simultaneously alter dozens of pathways. Such reductionism can confuse an associated biomarker with a necessary mediator. Stronger causal evidence requires perturbation of the proposed pathway, rescue experiments, dose-response relationships, cell-type-resolved analysis, and confirmation in models that reproduce the chronicity and cellular diversity of human disease. The most credible model is therefore not a catalogue of independent pathways but a network in which inflammatory licensing, secretory output, recipient-cell state, and tissue distribution jointly determine effect (31, 32, 34–43).

### 3.1 Neuroimmune and immunometabolic regulation

Chronic activation of microglia, astrocytes, infiltrating monocytes, and adaptive immune cells contributes to neuronal injury across AD, PD, ALS, MSA, and VaD. Licensed MSCs can release prostaglandin E2, transforming growth factor-beta, tumour necrosis factor-stimulated gene-6, indoleamine 2,3-dioxygenase, nitric oxide-related mediators, and adenosine-generating ectoenzymes. These signals may reduce pro-inflammatory cytokines, restrain inflammasome activation, promote reparative myeloid phenotypes, limit Th1 and Th17 responses, and expand regulatory T-cell activity. MSCs may also alter immune-cell metabolism by suppressing glycolytic inflammatory programmes, promoting oxidative phosphorylation, and depleting local tryptophan. Importantly, these effects are conditional: insufficient inflammatory licensing can yield weak potency, whereas severe or chronic inflammation may impair cell survival and secretory function (31, 32, 34–43).

Microglial polarisation is often simplified into pro-inflammatory M1 and reparative M2 states, but human microglia occupy dynamic transcriptional continua shaped by age, brain region, protein aggregates, vascular injury, and systemic immunity. Single-cell studies have identified disease-associated microglia (DAM) together with interferon-responsive, proliferative, lipid-handling, and other context-dependent programmes, emphasising that a change in a few M1/M2 markers cannot establish restoration of a beneficial state (336, 337). These states are not intrinsically protective or pathogenic across all stages: the same programme may support debris clearance early yet contribute to chronic inflammatory injury when sustained. Accordingly, prolonged indiscriminate immunosuppression could impair surveillance, clearance, or antimicrobial defence. The therapeutic objective should be selective resolution of maladaptive inflammation whilst preserving protective functions. Spatial transcriptomics, single-cell profiling, and lineage-specific perturbation should therefore determine whether MSCs or MSC-EVs reproducibly shift defined microglial states in human-relevant models, whether those changes precede neuronal protection, and whether the effect is product- and disease-stage specific. Similar precision is required for adaptive immunity, where effects on regulatory T cells, Th17 cells, B cells, and antigen-presenting cells may differ according to product licensing and disease context (35, 37, 38, 41, 42, 336, 337).

### 3.2 Neurotrophic support, proteostasis, and mitochondrial rescue

The MSC secretome contains brain-derived neurotrophic factor, glial cell line-derived neurotrophic factor, nerve growth factor, insulin-like growth factor-1, hepatocyte growth factor, vascular endothelial growth factor, anti-apoptotic proteins, and extracellular-matrix regulators. Together, these mediators can stabilise mitochondrial membranes, reduce caspase activation, support axonal and synaptic maintenance, and enhance endogenous repair. EV cargo can include microRNAs, mRNAs, proteins, lipids, and enzymes that influence autophagy, lysosomal function, oxidative stress, and pathological protein handling. Experimental evidence also supports transfer of functional mitochondria through tunnelling nanotubes or vesicular mechanisms, potentially restoring bioenergetics in injured neural cells. The frequency and quantitative importance of mitochondrial transfer in humans, however, remain uncertain. Most direct demonstrations derive from acute injury paradigms or short-term co-culture systems, whereas longitudinal evidence in chronic neurodegenerative models—and especially in patients—remains sparse. Mitochondrial transfer should therefore be treated as a plausible, context-dependent mechanism rather than a general explanation for MSC efficacy (31, 32, 34–43, 236).

Neurotrophic activity also demands pharmacological scrutiny. Factors such as BDNF, GDNF, NGF, IGF-1, VEGF, and HGF have short half-lives, act through concentration-dependent and sometimes opposing pathways, and may not reach vulnerable CNS regions after systemic administration. Measurements in conditioned medium cannot be assumed to represent *in vivo* exposure. Furthermore, trophic support may preserve stressed neurons but cannot restore cells that have been lost or correct a continuously active genetic or proteopathic insult. Future studies should quantify the active concentration delivered to target tissue, identify responsive neural populations, and test whether neuroprotection persists after product withdrawal. These requirements are particularly important when engineered MSCs overexpress potent factors, because supraphysiological signalling can create off-target vascular, proliferative, or nociceptive effects (34, 36, 39, 40, 43).

Proteostasis deserves similar caution. Enhanced autophagy, lysosomal activity, or proteasomal clearance is frequently inferred from a small set of markers measured at one time point. Increased autophagosome formation, for example, may reflect improved flux or impaired degradation. Studies of amyloid-beta and tau (70–90), alpha-synuclein (91–98), mutant huntingtin (99–121), or misfolded SOD1 (122–144) should distinguish reduced production, increased clearance, redistribution, and masking of toxic species. They should also determine whether the intervention affects soluble oligomers, insoluble aggregates, or cell-to-cell propagation, because these pools have different pathogenic significance. Mechanistic claims should be supported by dynamic flux assays and direct quantification of pathological species rather than by isolated signalling changes.

### 3.3 Neurovascular and blood-brain barrier effects

Neurodegeneration is increasingly understood as a disorder of the neurovascular unit as well as neurons. MSC-derived mediators may support endothelial survival, pericyte function, angiogenesis, tight-junction integrity, and cerebral microcirculation. These effects are particularly relevant to VaD but may also influence AD, PD, and MSA, in which blood-brain barrier disruption and impaired clearance of toxic proteins contribute to progression. Improved vascular integrity can reduce peripheral immune-cell entry and secondary inflammation, whilst angiogenic signalling may increase oxygen and nutrient delivery. Conversely, unregulated pro-angiogenic activity, variable vascular targeting, and poor tissue retention create safety and efficacy uncertainties.

The neurovascular unit provides a useful example of why disease context matters. In VaD, endothelial dysfunction and impaired perfusion are proximal drivers, making vascular repair a direct therapeutic objective. In AD or PD, vascular dysfunction may amplify protein accumulation and inflammation but may not be the initiating lesion. Angiogenesis is likewise not synonymous with functional repair: newly formed vessels must be stable, perfused, appropriately innervated, and integrated with pericytes and astrocytic end-feet. Consequently, vascular endpoints should include permeability, perfusion, oxygen extraction, barrier transport, and neurovascular coupling rather than vessel density alone. Product-specific procoagulant activity and the possibility of microvascular obstruction also require evaluation, particularly with intra-arterial or high-dose intravenous delivery (15, 34, 36, 39, 40, 43).

### 3.4 Cell-free products

MSC-EVs may reproduce selected parental-cell effects whilst offering lower embolic risk, easier cryostorage, and greater potential for engineering. Surface modification, cargo loading, and intranasal or other targeted delivery could enhance CNS exposure. Yet EV preparations are highly sensitive to culture conditions, isolation methods, purification, storage, and dose metrics. Particle number, protein content, lipid content, and source-cell equivalents are not interchangeable measures. Preparations can contain non-vesicular proteins, lipoproteins, or process-related contaminants, and potency may not correlate with particle concentration. Accordingly, EVs should be developed as defined biological products with identity, purity, cargo, functional potency, stability, biodistribution, and toxicology specifications rather than as loosely characterised derivatives. The integrated mechanisms of MSC and MSC-EV neuroprotection are shown in **Figure 2**.

**Figure 2:**
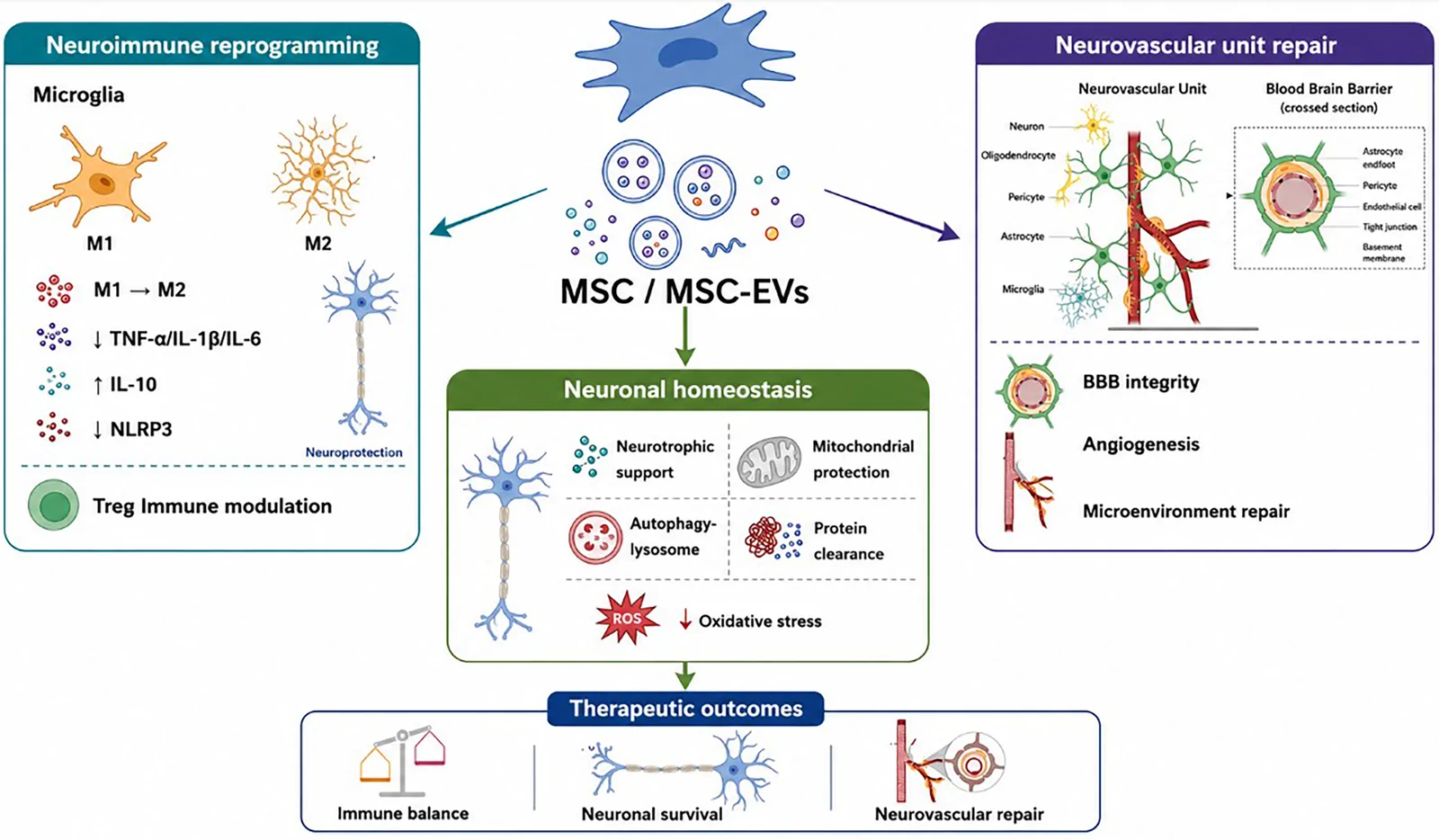
Core mechanisms of MSC/MSC-EV-mediated neuroprotection. This schematic summarises the principal neuroprotective mechanisms mediated by mesenchymal stromal cells (MSCs) and MSC-derived extracellular vesicles (MSC-EVs) in neurodegenerative diseases. MSC/MSC-EVs act through three interconnected pathways: (1) neuroimmune reprogramming, characterised by attenuation of maladaptive microglial and astrocytic inflammatory states, reduced TNF-α, IL-1β, IL-6, and NLRP3 inflammasome activity, and enhanced regulatory and inflammation-resolving signals; (2) maintenance of neuronal homeostasis through neurotrophic support, mitochondrial protection, autophagy-lysosome regulation, improved protein clearance, and attenuation of oxidative stress; and (3) neurovascular-unit repair, including restoration of blood-brain barrier integrity, support of endothelial cells and pericytes, and improvement of local perfusion and vascular stability. Collectively, these mechanisms may promote immune balance, neuronal survival, and neurovascular integrity. MSC, mesenchymal stromal cell; MSC-EV, mesenchymal stromal cell-derived extracellular vesicle; TNF-α, tumour necrosis factor-alpha; IL, interleukin; NLRP3, NOD-like receptor family pyrin domain containing 3; BBB, blood-brain barrier.

A further conceptual challenge is that EVs are not miniature versions of MSCs. They represent one component of a broader secretome and lack the capacity to sense the host environment and dynamically alter secretion after administration. This loss of responsiveness may improve standardisation but remove an important feature of living-cell therapy. Conversely, EV cargo can be enriched, depleted, or redirected during manufacturing, enabling a more conventional dose-response relationship. Comparative studies should therefore ask which biological functions require viable, environmentally responsive cells and which can be reproduced by a stable acellular product. Direct comparisons using matched parental cells, secretome fractions, and purified EVs are more informative than separate experiments performed with unrelated preparations (4, 10, 12, 14, 36, 43).

Viewed side by side, the two platforms solve different translational problems. Intact MSCs provide dynamic inflammatory licensing, a broader secretome, cell–cell interactions, and—at least experimentally—the possibility of organelle transfer; their liabilities include post-thaw viability loss, pulmonary trapping after intravenous infusion, procoagulant activity, alloimmune recognition with repeat dosing, and marked donor- and process-dependent variability. MSC-EVs avoid viable-cell proliferation and microvascular cell trapping, are more amenable to repeated administration, cryostorage, cargo engineering, and surface targeting, and can be characterised with particle-based analytics. However, they lose adaptive sensing, are rapidly cleared by the mononuclear phagocyte system, lack a universally accepted dose metric, and remain vulnerable to co-isolated proteins/lipoproteins, cargo drift, and batch-to-batch variability. Thus, EVs are not intrinsically ‘more standardised’ than cells; they become more standardisable only when source-cell state, isolation, purity, cargo, stability, biodistribution, and mechanism-linked potency are controlled as rigorously as the parent cell product (4, 10, 12, 14, 36, 43).

### 3.5 Ageing biology as a determinant of MSC efficacy

Ageing is not merely a background variable in neurodegeneration; it changes the therapeutic target. Inflammaging, immunosenescence, mitochondrial dysfunction, impaired autophagy, endothelial senescence, blood-brain barrier leakage, and reduced neuroplasticity can alter both the need for and the response to MSC-derived signals. A product that suppresses acute inflammation in a young experimental model may perform differently in an older recipient with chronic myeloid activation, vascular comorbidity, impaired clearance pathways, and reduced structural reserve. Age should therefore be incorporated into model selection, biodistribution studies, pharmacodynamic sampling, and clinical stratification rather than treated only as a demographic covariate (75, 98, 145, 146).

Ageing also affects the product itself. Adult donor age, metabolic disease, inflammatory exposure, replicative expansion, and cryopreservation can promote cellular senescence, reduce proliferative fitness, alter extracellular-vesicle cargo, and shift the secretome towards a senescence-associated secretory phenotype (SASP) enriched in inflammatory and matrix-remodelling mediators. In an autologous strategy for an older patient, this creates a biologically important paradox: the product is derived from the same aged, metabolically stressed milieu that the therapy is intended to correct, and a high senescence burden could attenuate immunoregulatory or trophic potency or, in an unfavourable context, reinforce neuroinflammation. Candidate sources should consequently be compared under a common manufacturing process, with release criteria that include senescence burden, mitochondrial fitness, procoagulant activity, and indication-relevant potency rather than identity markers alone (44, 45, 49, 50).

The choice between an autologous aged product and an allogeneic young-donor product is therefore a therapeutic and ethical trade-off rather than a categorical preference. Autologous MSCs minimise donor–recipient disparity and preserve patient-specific provenance, but manufacturing is slower and may transmit age-, disease-, medication-, or metabolic-state-related dysfunction. Young-donor allogeneic products can be banked, batch-selected for potency, and delivered rapidly, which is attractive in geriatric disorders such as AD and VaD; however, they require stringent donor governance, comparability across banks, testing for anti-donor responses and repeat-dose immunogenicity, and transparent justification of donor selection. Trials should prospectively compare these strategies using the same potency framework rather than treating ‘autologous’ or ‘allogeneic’ as a surrogate for quality.

## 4 Disease-specific evidence

The therapeutic landscape across six major neurodegenerative diseases and selected rare disorders is summarised in **Figure 3**. Although the same broad mechanisms recur, each disorder presents a different dominant pathology, therapeutic window, clinical trajectory, and endpoint structure. The rare-disease panel is included as an exploratory boundary case and does not imply shared pathogenesis, equivalent therapeutic maturity, or a common evidentiary standard. Disease-specific development is therefore essential.

**Figure 3:**
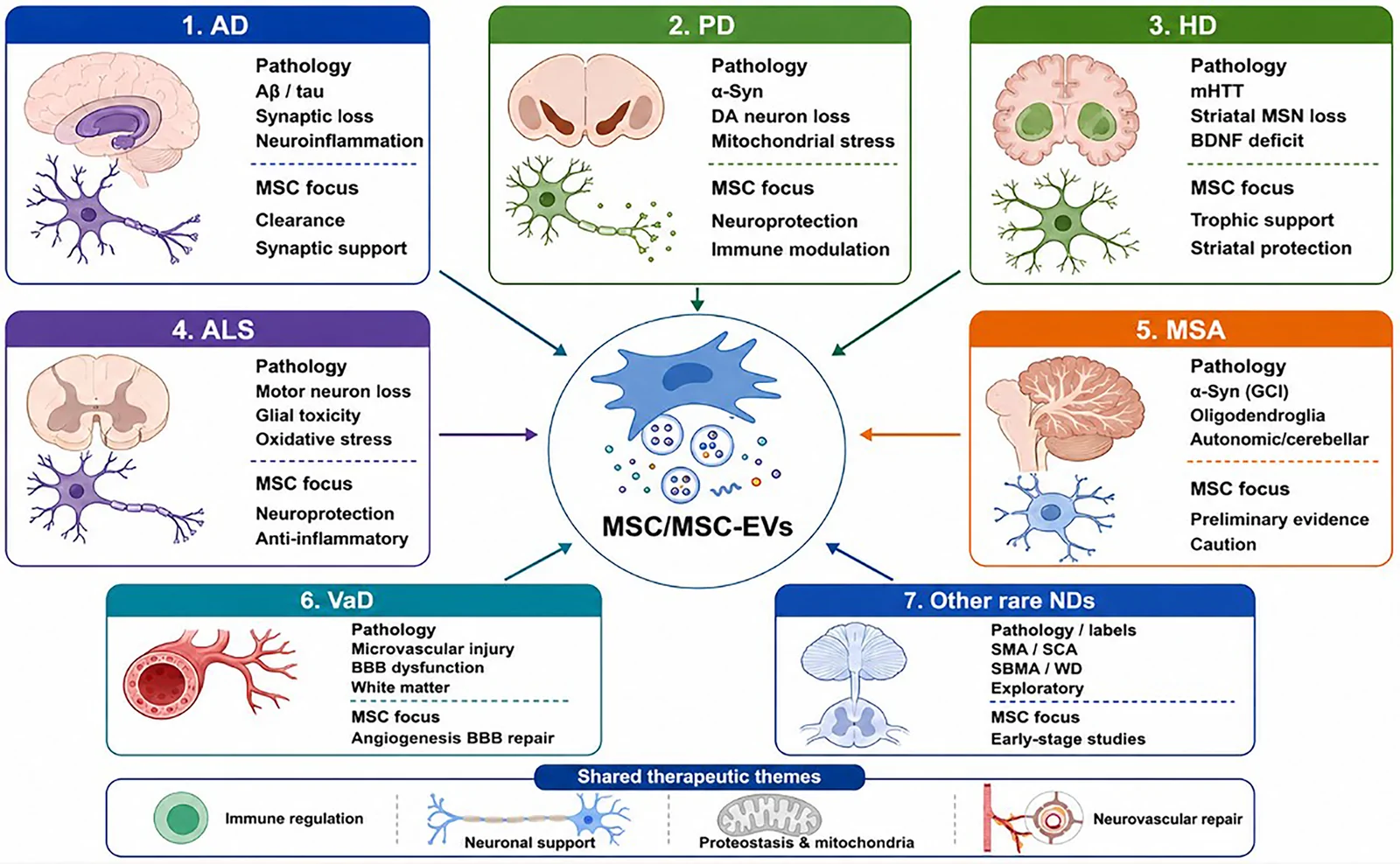
Disease-specific application landscape of MSC therapy across age-related and selected rare neurodegenerative disorders. This schematic summarises the current applications of mesenchymal stromal cells (MSCs) and MSC-derived extracellular vesicles (MSC-EVs) across seven representative disease contexts: Alzheimer’s disease (AD), Parkinson’s disease (PD), Huntington’s disease (HD), amyotrophic lateral sclerosis (ALS), multiple system atrophy (MSA), vascular dementia (VaD), and an exploratory group of other rare neurodegenerative disorders, including spinal muscular atrophy (SMA), spinocerebellar ataxia (SCA), spinal and bulbar muscular atrophy (SBMA), and Wilson’s disease (WD). Each disease module highlights dominant pathological features and the most plausible MSC-mediated therapeutic roles, including immunomodulation, trophic support, synaptic preservation, proteostasis, mitochondrial maintenance, and neurovascular repair. Evidence in MSA and the rare-disease group remains preliminary and should be interpreted cautiously; the grouping does not imply shared pathogenesis or comparable clinical maturity. The shared-mechanism panel emphasises that convergent biological activities still require disease-specific product selection, therapeutic timing, standard-of-care integration, and outcome measures. MSC, mesenchymal stromal cell; MSC-EV, mesenchymal stromal cell-derived extracellular vesicle; AD, Alzheimer’s disease; PD, Parkinson’s disease; HD, Huntington’s disease; ALS, amyotrophic lateral sclerosis; MSA, multiple system atrophy; VaD, vascular dementia; ND, neurodegenerative disease; SMA, spinal muscular atrophy; SCA, spinocerebellar ataxia; SBMA, spinal and bulbar muscular atrophy; WD, Wilson’s disease; BBB, blood-brain barrier; GCI, glial cytoplasmic inclusion; DA, dopaminergic; MSN, medium spiny neuron; α-Syn, α-synuclein; mHTT, mutant huntingtin; BDNF, brain-derived neurotrophic factor.

### 4.1 Alzheimer’s disease

AD combines amyloid-beta accumulation, tau pathology, synaptic failure, mitochondrial dysfunction, vascular impairment, and sustained neuroinflammation. MSCs and MSC-EVs have been reported to reduce amyloidogenic processing, increase amyloid-degrading enzymes, modulate tau-related signalling, and promote autophagic or lysosomal clearance in experimental systems. They can also suppress activated microglia, increase neurotrophic support, protect hippocampal neurons, and improve synaptic plasticity. These multimodal effects are appealing because intervention at a single pathological target has produced limited clinical benefit in many settings. However, mechanistic findings vary by model, and improvements in behavioural tests do not necessarily demonstrate durable modification of human AD pathology (70–90, 147–151).

The AD literature illustrates both the promise and the interpretive hazards of multimodal therapy. In transgenic and chemically induced models, MSC products have been associated with reduced BACE1 activity, increased neprilysin-mediated amyloid degradation, altered glycogen synthase kinase-3beta and PI3K-AKT signalling, attenuation of tau phosphorylation, and improved autophagic flux. These effects converge on lower synaptic stress and improved behavioural performance. However, many models express one dominant pathological feature over a compressed time course and do not recapitulate the mixed amyloid, tau, vascular, inflammatory, and age-related pathology of sporadic human AD. A treatment that improves memory in a young transgenic mouse may therefore reflect acute anti-inflammatory or behavioural effects rather than durable modification of human neurodegeneration (70–90, 147–151).

Preclinical studies generally report improved learning and memory, reduced inflammatory signalling, and lower pathological-protein burden after administration of bone marrow-, adipose-, umbilical cord-, dental-, or olfactory mucosa-derived products. Outcomes depend on dose, route, timing, cell source, and disease model. Treatment before extensive neuronal loss usually produces larger effects than late intervention, suggesting that MSC-based therapies are more likely to preserve vulnerable networks than reconstruct destroyed circuitry. Engineered EVs and preconditioned MSCs can enhance brain targeting or expression of neuroprotective cargo, but comparisons are difficult because manufacturing and outcome measures are inconsistent (70–90, 147, 149, 151).

Timing is likely to be decisive. Once widespread neuronal loss, network disconnection, and cerebral atrophy are established, paracrine support cannot reconstruct the original circuitry. Preclinical treatment is commonly initiated before or soon after pathological onset, whereas patients enter trials after years of silent progression. This mismatch can inflate expectations and may explain why modest signals are concentrated in mild disease. A rational programme would define a therapeutic window using amyloid and tau biomarkers, neurodegeneration markers, inflammatory signatures, and structural reserve, then test whether target engagement changes the slope of decline. The relevant claim is not reversal of dementia but preservation of function in a biologically selected population (146, 152–163).

Clinical evidence remains preliminary. Early-phase trials of intact MSCs or MSC-derived products have mainly assessed safety and feasibility, with variable signals in cognition, composite disease scores, imaging, or inflammatory biomarkers. Apparent benefit is more plausible in mild or early-stage disease, whereas established moderate-to-severe neurodegeneration is unlikely to be reversed. Small samples, short follow-up, differing background therapies, and reliance on cognitive scales limit interpretation. Objective measures of amyloid and tau burden, neurodegeneration, cerebral metabolism, inflammation, and atrophy should be integrated with function and quality of life. Claims of marked improvement require particular caution because practice effects, natural variability, and caregiver expectations can influence behavioural outcomes (152, 154, 156–158, 160–163).

Clinical interpretation also requires separation of product effects from procedural and contextual effects. Intracerebral or intraventricular administration may generate inflammatory or placebo responses, whilst repeated cognitive testing can produce practice effects. Background use of cholinesterase inhibitors, memantine, anti-amyloid therapies, vascular-risk management, and rehabilitation can influence trajectories. Small trials are especially vulnerable to baseline imbalance and selective reporting of favourable domains. Future studies should pre-specify a limited endpoint hierarchy, use centralised imaging and biomarker analysis, report all randomised participants, and publish neutral outcomes. Demonstration of reduced inflammatory markers without parallel evidence of slowed neurodegeneration should be viewed as pharmacodynamic activity rather than proof of clinical efficacy (152–154, 156–158, 160–165).

Cell-free AD programmes face additional challenges. Intranasal delivery is attractive because it is non-invasive and potentially repeatable, but deposition within the nasal cavity, mucociliary clearance, age-related mucosal change, and variability in device technique can produce inconsistent exposure. Systemically administered EVs are rapidly distributed to liver, spleen, and lung, and claims of blood-brain barrier penetration often derive from labelled-particle experiments vulnerable to dye transfer or free-label artefact. Human studies should therefore incorporate validated biodistribution methods, dose recovery, and pharmacodynamic markers in cerebrospinal fluid or imaging. Without such evidence, improved cognition cannot be confidently attributed to delivery of therapeutic cargo to the brain (73, 85, 88, 153, 155, 163, 164, 166–172).

AD development should prioritise biomarker-defined early populations, including stratification by pathological confirmation, inflammatory status, vascular burden, genotype, and stage. Product selection should match the hypothesised mechanism: an immunoregulatory product may be suited to an inflammation-enriched phenotype, whereas an engineered EV may be designed for pathological-protein clearance or trophic support. Long-term trials must distinguish transient symptomatic effects from slower structural or biomarker progression and should include delayed adverse events, immune responses, and tumour surveillance. Standardised manufacturing, scalable production, and rational combinations with anti-amyloid, anti-tau, anti-inflammatory, or neuroprotective therapies may be more realistic than stand-alone use (146, 153, 155, 159, 164–172).

The evidentiary benchmark should also reflect the changing AD treatment landscape. As disease-specific drugs become available, an MSC product will need to demonstrate incremental value rather than superiority to placebo in therapeutic isolation. Relevant roles might include treating patients with persistent inflammatory or vascular dysfunction despite pathological-protein-directed therapy, reducing treatment-related neuroinflammation, or preserving neural function through an orthogonal mechanism. Add-on trials should be stratified by concomitant disease-modifying treatment and designed to identify pharmacodynamic interaction. This positioning is more credible than presenting MSCs as an alternative capable of simultaneously replacing all established and emerging therapies (152–154, 156–158, 160–163, 165).

### 4.2 Parkinson’s disease

PD is characterised by degeneration of nigrostriatal dopaminergic neurons, alpha-synuclein accumulation, mitochondrial impairment, oxidative stress, and neuroinflammation. The original goal of differentiating MSCs into dopamine-producing neurons has largely yielded to a paracrine model. MSCs and EVs can reduce microglial inflammatory activity, inhibit inflammasome-related pathways, increase trophic support, and improve antioxidant defences. Experimental studies also suggest effects on autophagy, endoplasmic-reticulum stress, alpha-synuclein handling, mitochondrial biogenesis, and intercellular mitochondrial transfer. These mechanisms could protect residual neurons but have not convincingly been shown to eliminate established Lewy pathology or restore complex basal-ganglia circuitry (91–98, 173–182).

In PD, preservation of residual dopaminergic neurons is biologically more plausible than replacement of the nigrostriatal system. The disease extends beyond the substantia nigra to autonomic, olfactory, limbic, cortical, and peripheral networks, and alpha-synuclein pathology propagates across anatomically connected regions. A focal graft cannot readily address this distributed process, whilst systemic immunomodulation may affect only one component of pathogenesis. MSC-mediated suppression of NLRP3 activation, oxidative stress, and mitochondrial failure could slow secondary injury, but evidence that these products interrupt the templated spread of alpha-synuclein or remove mature Lewy pathology is limited. Claims of disease modification should therefore require longitudinal dopaminergic imaging, alpha-synuclein-related biomarkers when validated, and sustained change after controlling for symptomatic medication (91–98, 173, 181).

Source-related differences are relevant. Bone marrow MSCs have the most extensive clinical experience but are affected by donor age and invasive collection. Umbilical cord and Wharton’s jelly products offer expansion and allogeneic-manufacturing advantages, adipose-derived MSCs permit abundant autologous collection but may be influenced by metabolic disease, and dental-derived cells have neural crest characteristics but limited yield. Direct comparisons under identical culture and assay conditions are rare. Thus, observed differences between studies may reflect donor selection, passage, media, cryopreservation, viability, or potency as much as tissue origin itself (183–198).

Autologous and allogeneic strategies entail different compromises. Autologous adipose or bone marrow products reduce donor-recipient immunological disparity but are manufactured from older patients whose cells may carry senescence, mitochondrial dysfunction, medication exposure, and metabolic abnormalities. Allogeneic perinatal products can be banked and standardised but may be cleared more rapidly after repeated dosing and require donor-to-donor comparability. Cryopreserved off-the-shelf products improve logistics, yet post-thaw dysfunction can alter homing, viability, and secretory activity. Trials should report fresh versus cryopreserved status, time from thaw to administration, viable-cell dose, passage, senescence markers, and functional potency so that clinical outcomes can be interpreted as properties of a defined product rather than of the generic cell class (183, 186–191, 193–196, 198).

Delivery remains a central constraint. Stereotactic injection achieves local exposure but is invasive and cannot address diffuse pathology. Intravenous administration is practical and repeatable, although pulmonary retention results in very low brain delivery; benefits may therefore arise partly from peripheral immune regulation. Intrathecal and intra-arterial routes increase CNS proximity but add procedural risk. Intranasal administration is promising for cells or EVs because it may use olfactory and trigeminal pathways, yet human dose distribution, mucosal clearance, and reproducibility are uncertain. Focused-ultrasound-assisted barrier opening and targeted carriers remain experimental (199–204).

Clinical studies broadly support short-term tolerability, with fever, headache, or procedure-related events more common than severe treatment-related toxicity. Open-label studies have described changes in motor scales, medication requirements, non-motor symptoms, or inflammatory biomarkers, but controlled trials have produced smaller and less consistent effects. Placebo response, fluctuating symptoms, dopaminergic medication timing, disease subtype, and baseline inflammatory status complicate assessment. Trials should incorporate blinded motor ratings, wearable measures, imaging, fluid biomarkers, medication-adjusted outcomes, and follow-up sufficient to test disease modification rather than transient symptom change (205–208).

The discrepancy between open-label and controlled PD studies deserves emphasis. Motor symptoms vary with medication timing, sleep, anxiety, assessor expectations, and natural fluctuations. Improvements in the Unified Parkinson’s Disease Rating Scale after an invasive or highly anticipated treatment can therefore overestimate biological effect. Objective measures - home-based wearable monitoring, timed motor tasks, quantitative gait analysis, medication-adjusted assessments, and imaging - should complement clinician-rated scales. Non-motor outcomes should be selected according to the proposed mechanism rather than surveyed indiscriminately. A trial designed around immunomodulation, for example, should show a coherent sequence from immune target engagement to neuronal preservation and then to clinical benefit (190, 191, 195, 196, 205–208).

PD also requires distinction between neuroprotection and dopaminergic symptom enhancement. Engineered cells capable of producing dopamine or secreting high levels of trophic factors may improve motor behaviour without altering alpha-synuclein pathology or the rate of neuronal loss. Such a product could still be clinically useful, but it should be described as a biological symptomatic therapy unless durable preservation is demonstrated. Conversely, an anti-inflammatory product may slow degeneration without producing an immediate change in motor scores. Trial duration, endpoint selection, and medication adjustment must therefore correspond to the expected temporal profile of the mechanism (190, 191, 205–215).

Next-generation PD strategies include hypoxic or pharmacological preconditioning, genetic enhancement of BDNF, GDNF, VEGF, or dopamine-related pathways, and engineered EVs carrying regulatory RNAs or drugs. These approaches may improve potency and targeting but introduce additional manufacturing, genomic, biodistribution, and regulatory risks. Development should progress only when the enhanced function is measurable with a validated potency assay and when exposure can be linked to a defined biological and clinical endpoint (209–225).

### 4.3 Huntington’s disease

HD is a monogenic disorder caused by expanded CAG repeats in HTT, leading to mutant huntingtin toxicity, striatal medium spiny neuron loss, impaired trophic transport, mitochondrial dysfunction, proteostasis failure, and neuroinflammation. MSC-based therapy cannot correct the mutation unless combined with gene-silencing or editing technologies, but it may protect vulnerable networks and modify the tissue environment. Experimental studies report increased BDNF signalling, reduced microglial activation, improved oxidative and mitochondrial homeostasis, and altered autophagy or glymphatic clearance. Conditioned medium and EVs reproduce several effects, supporting a paracrine rather than replacement mechanism (99–121).

Because the causal mutation is known, HD provides a stringent test of therapeutic positioning. MSCs cannot suppress mutant huntingtin production unless engineered to deliver gene-silencing cargo; unmodified products can only modify downstream stress responses. Their value may lie in increasing BDNF support, stabilising mitochondrial and redox homeostasis, modulating glial inflammation, and improving the extracellular environment in which vulnerable medium spiny neurons function. These effects could extend neuronal resilience but would be expected to diminish whilst mutant huntingtin remains continuously expressed. The most compelling strategy is therefore complementary: combine a causal intervention with a transient biological product that protects tissue during the period required for genetic therapy to act (99–121).

HD offers a distinctive opportunity for preventive or early intervention because genetically at-risk individuals can be identified before overt symptoms. Engineered MSCs overexpressing BDNF or carrying RNA-interference cargo have produced stronger preclinical effects than unmodified cells, and pharmacological preconditioning may enhance survival without permanent genomic modification. MSCs could also serve as transient carriers or microenvironmental adjuncts to antisense oligonucleotides, gene therapy, or small molecules. Nonetheless, rodent toxin models and rapidly progressive transgenic models do not reproduce the full time course of human HD, and effect sizes may be exaggerated by small, non-blinded studies (226–239).

The long premanifest phase creates an opportunity unavailable in many sporadic disorders, but it also raises ethical and methodological challenges. Treating mutation carriers before symptoms requires exceptional long-term safety and a credible biomarker of benefit, because clinical conversion may take years. Short-term changes in inflammation or neurotrophic markers cannot justify an invasive intervention in otherwise functional individuals. Early manifest cohorts may offer a more practical initial population, provided that progression is quantified using composite motor, cognitive, functional, imaging, and mutant huntingtin measures. Trials should be powered for a change in disease trajectory and should avoid interpreting transient symptomatic improvement as modification of the genetic disorder (226–250).

Model selection is especially important in HD. Toxin-based striatal lesions reproduce neuronal injury but not the continuous production and systemic consequences of mutant huntingtin. Transgenic models differ in repeat length, fragment expression, progression speed, and behavioural phenotype. A therapeutic effect confined to acute lesion models provides limited support for human translation. Evidence should converge across full-length genetic models, patient-derived cells or organoids, and *in vivo* measures of mutant huntingtin, synaptic function, and neurodegeneration. The intervention should also be tested alongside contemporary huntingtin-lowering strategies, because the relevant clinical question is whether it adds value to causal therapy rather than whether it outperforms no treatment (99, 100, 102, 103, 105, 107, 110, 112, 113, 116, 117, 119–121, 240–250).

Clinical translation is limited by uncertain long-term safety, invasive delivery, product variability, and the need for prolonged observation in slowly evolving disease. Intracerebral administration offers local targeting but carries surgical risk; systemic delivery is safer but inefficient; intranasal delivery remains investigational. Future trials should prioritise well-characterised premanifest or early manifest cohorts, validated mutant huntingtin and neurodegeneration biomarkers, quantitative motor and cognitive measures, and combination strategies that pair causal genetic intervention with MSC-mediated trophic or immunological support (240–250).

### 4.4 Amyotrophic lateral sclerosis

ALS causes progressive loss of upper and lower motor neurons and is biologically heterogeneous with respect to genetics, site of onset, progression rate, cortical involvement, and inflammatory phenotype. MSCs are unlikely to replace motor neurons or reconstruct long axonal projections. Their rationale rests on secretion of BDNF, GDNF, IGF-1, VEGF, and other trophic mediators; modulation of microglia, astrocytes, and peripheral immunity; reduction of oxidative injury; and delivery of protective EV cargo. These mechanisms have repeatedly delayed functional decline or prolonged survival in SOD1-based animal models, but most human ALS is sporadic and differs substantially from these experimental systems (122–144).

The ALS evidence base is particularly instructive because strong preclinical rationale has repeatedly failed to yield consistent pivotal-trial benefit. Motor-neuron degeneration is accompanied by astrocytic toxicity, microglial activation, impaired axonal transport, mitochondrial dysfunction, excitotoxicity, and systemic metabolic change. MSC products can influence several of these pathways, and engineering cells to secrete neurotrophic factors increases biological activity. Yet the anatomical challenge is formidable: upper and lower motor neurons are distributed across cortex, brainstem, and spinal cord, and each has long axons whose neuromuscular connections may already be irreversibly lost. Local delivery can create high exposure in one compartment without reaching the full motor system, whereas systemic delivery provides uncertain CNS concentrations (122, 123, 125–129, 131–136, 138, 139, 141–143).

Clinical development illustrates the gap between early signals and confirmatory evidence. Phase I studies of intraspinal, intrathecal, or intravenous autologous and allogeneic products generally established feasibility. Some phase II studies suggested slower decline in selected patients or temporary biomarker changes. However, larger controlled trials, including programmes using neurotrophic-factor-secreting autologous MSCs, have not consistently met primary endpoints. *Post hoc* responder analyses may generate hypotheses but cannot replace prospectively defined stratification (251–268).

The phase III MSC-NTF (NurOwn) programme provides a concrete example. In the randomised, double-blind, placebo-controlled trial, 196 participants were randomised and 189 received at least one treatment; the autologous neurotrophic-factor-secreting product was administered intrathecally at weeks 0, 8, and 16. The prespecified responder endpoint was not met: 32.6% of MSC-NTF-treated participants and 27.7% of placebo-treated participants achieved the defined improvement in ALSFRS-R slope (odds ratio 1.33; P = 0.45), and the main secondary clinical endpoints were also neutral, despite clear changes in several cerebrospinal-fluid pharmacodynamic biomarkers (124). The trial therefore separates biological target engagement from clinical validation. Its investigators highlighted advanced baseline disease, heterogeneity, and ALSFRS-R floor effects as important limitations; the programme also leaves unresolved whether autologous product potency, compartmental CNS exposure, and repeated intrathecal distribution were sufficiently consistent for a multifocal motor-system disease. *Post hoc* signals in less advanced subgroups are hypothesis-generating, not confirmatory. This failure does not establish class-wide inefficacy, but it shows why potency, exposure, prospective stratification, and endpoint sensitivity must be linked before progression to a pivotal trial.

The distinction between target engagement and efficacy is essential. Changes in cerebrospinal fluid cytokines or neurotrophic factors can confirm that an administered product is biologically active, but they do not establish preservation of motor units. Conversely, a global functional scale may miss a biologically meaningful effect in a subgroup or over a short interval. An integrated trial should therefore demonstrate product potency before dosing, exposure or pharmacodynamic activity after dosing, reduction in neuronal injury markers such as neurofilament, preservation of respiratory or electrophysiological function, and a clinically meaningful effect on function or survival. Failure at any step should refine the mechanism rather than prompt unstructured repetition with another cell dose (251–274).

ALS trial populations may also require pathway-specific selection. Neuroinflammatory profiles, genetic variants, lower-motor-neuron predominance, bulbar onset, respiratory involvement, and progression rate can influence both mechanism and measurable response. A broadly immunomodulatory product may be most effective in a subgroup with active immune dysregulation but less useful in rapidly progressive disease dominated by irreversible axonal loss. Stratified randomisation and prespecified treatment-by-biomarker interactions can test this hypothesis. Importantly, biomarker enrichment should not become a retrospective explanation for a failed trial; assays and thresholds need analytical validation before they determine eligibility (251–268, 275–280).

Several factors plausibly contributed to failure. ALS trials often enrol biologically dissimilar patients and use dosing regimens inherited from safety studies rather than pharmacological optimisation. The ALS Functional Rating Scale-Revised is clinically meaningful but may be insensitive to short or heterogeneous treatment effects. Autologous cells from older or rapidly progressive patients can show impaired proliferation and secretory function, whilst repeated manufacture introduces variability. Intravenous delivery provides low CNS exposure; intrathecal delivery increases proximity but does not ensure distribution throughout the motor system; intraspinal delivery is invasive and focal. Product identity and potency have rarely been linked to individual exposure or response (269–274, 281–284).

Autologous manufacturing introduces an additional selection problem in ALS. Patients with rapidly progressive disease may have insufficient time for product manufacture and may also contribute cells with impaired proliferative or secretory capacity. Those who successfully receive treatment can therefore represent a healthier, slower-progressing subset, creating bias in uncontrolled studies. Allogeneic products permit immediate treatment and batch testing but shift the problem towards immunological compatibility and repeat dosing. Prospective trials should account for manufacture-related attrition, use intention-to-treat analyses from the point of randomisation, and report the proportion and characteristics of patients who fail product release (251–268, 275–280).

Future ALS programmes should use biomarker-guided enrichment, including inflammatory, neurofilament, genetic, and progression-rate profiles; define dose and schedule using biodistribution and pharmacodynamic data; and combine functional outcomes with neurofilament, respiratory, electrophysiological, and survival measures. Allogeneic or engineered products may avoid dysfunction in autologous cells, whilst EV-based therapy may improve standardisation and repeat dosing. These advantages remain theoretical until robust manufacturing and controlled clinical efficacy are demonstrated (275–280).

### 4.5 Multiple system atrophy

MSA is a rapidly progressive alpha-synucleinopathy involving autonomic, parkinsonian, cerebellar, and pyramidal systems. MSCs may suppress microglial activation, provide trophic support to neurons and oligodendrocytes, improve vascular or barrier function, and reduce secondary oxidative injury. Transgenic and toxin-based models have shown functional and pathological improvements, but they capture only parts of human MSA and use heterogeneous cell products and routes (285–294).

MSA also exposes the limitations of relying on an umbrella diagnosis. Parkinsonian and cerebellar phenotypes differ in regional degeneration and progression, whilst autonomic failure can dominate disability and mortality. A treatment delivered to the arterial circulation, cerebrospinal fluid, or systemic compartment may reach these systems unevenly. Early reports of slowed decline or reduced cortical atrophy are hypothesis-generating, but small samples and route-specific procedures make replication essential. Future studies should stratify phenotype, quantify autonomic as well as motor outcomes, and determine whether biomarker evidence of glial alpha-synuclein pathology or neuroinflammation identifies a responsive window (287, 295, 296).

The rapid progression of MSA creates both an opportunity and a risk. A disease-modifying effect may be detected over a shorter interval than in AD, but deterioration can also magnify the impact of missing data and baseline imbalance. Autonomic instability increases procedural risk for intra-arterial or intrathecal delivery, and apparent functional stabilisation may be influenced by supportive treatment. Trials should therefore include central adjudication, robust handling of death and dropout, and outcomes spanning autonomic function, mobility, activities of daily living, and survival. Imaging should be interpreted as supportive rather than definitive unless changes are linked to clinical preservation (287, 295–304).

Clinical studies have explored intra-arterial, intravenous, and intrathecal administration. Early open-label and small randomised studies reported acceptable short-term safety and possible slowing of functional decline or cortical atrophy, whilst later dose-escalation work supported intrathecal feasibility. Systematic reviews nevertheless identify small samples, variable controls, inconsistent outcome measures, and limited replication. The main unresolved issues are whether any effect is disease modifying, which route provides adequate distribution without unacceptable risk, and whether intervention must occur before extensive multisystem degeneration. Future trials require harmonised products, early-stage cohorts, autonomic and motor endpoints, imaging and fluid biomarkers, and longer follow-up (295–304).

### 4.6 Vascular dementia

VaD encompasses heterogeneous cognitive syndromes caused by large-vessel disease, small-vessel disease, strategic infarction, haemorrhage, or chronic hypoperfusion. MSCs are attractive because they can address vascular and neural components simultaneously. In experimental models, they promote endothelial survival, angiogenic signalling, pericyte and tight-junction support, reduce inflammatory activation, and enhance synaptic plasticity. EV cargo has been linked to neurovascular repair and reduced white-matter injury, whilst combinations with pharmacological or physical interventions may produce additive effects (305–314).

The heterogeneity of VaD requires a move away from syndromic enrolment. A patient with strategic infarction, diffuse small-vessel disease, cerebral amyloid angiopathy, or mixed AD-vascular pathology may have the same cognitive label but a different therapeutic target. MSC-mediated endothelial and barrier repair is most coherent in active microvascular dysfunction with salvageable white matter, whereas established multi-infarct tissue loss offers little substrate for regeneration. Trials should use vascular imaging, perfusion, barrier permeability, white-matter integrity, and vascular-risk profiles to define subgroups and should maintain rigorous control of blood pressure, diabetes, lipids, antithrombotic therapy, and rehabilitation (145, 305, 315–322).

Preclinical VaD studies should likewise move beyond short-term behavioural rescue after acute hypoperfusion. Human cerebral small-vessel disease evolves over years and is shaped by endothelial senescence, blood-brain barrier leakage, impaired vasoreactivity, inflammation, and recurrent microscopic injury. A persuasive model should use aged animals with vascular comorbidity, clinically relevant treatment timing, and longitudinal imaging. It should test whether MSC products improve perfusion and barrier function without promoting unstable angiogenesis or thrombosis. Cognitive benefit should be accompanied by preservation of white matter, axonal connectivity, and neurovascular coupling (305–314).

The evidence is mainly preclinical. Behavioural improvement can be confounded by model selection and testing bias, and increased vessel density does not necessarily indicate functional microcirculation or durable cognitive benefit. Ageing, diabetes, hypertension, and atherosclerosis may impair both host responsiveness and autologous cell potency. Cell and EV studies use inconsistent sources, doses, routes, and outcome measures. Human studies are small and generally uncontrolled, supporting feasibility rather than efficacy (145, 315–322). Development should focus on pathologically defined subtypes, particularly cerebral small-vessel disease with blood-brain barrier dysfunction; objective imaging of perfusion, white-matter integrity, and network connectivity; vascular and inflammatory biomarkers; and clinically meaningful cognition and daily function.

### 4.7 Selected rare neurodegenerative disorders

Exploratory MSC research extends to spinal muscular atrophy (SMA), spinocerebellar ataxias (SCAs), spinal and bulbar muscular atrophy (SBMA), and Wilson’s disease (WD), which are included in **Figure 3** as boundary cases rather than as a biologically uniform category. In SMA, preclinical extracellular-vesicle work and isolated clinical reports suggest possible support of motor neurons and peripheral tissues, but MSC products do not correct survival motor neuron deficiency and should not be interpreted as substitutes for disease-specific molecular therapy (323–325). In SCAs, experimental and early clinical studies suggest Purkinje-cell protection, trophic support, and possible functional stabilisation; however, the evidence is heterogeneous, mostly uncontrolled, and not sufficient to establish disease modification (326–329).

Evidence in SBMA is even less mature and primarily concerns cellular modelling rather than therapeutic administration (330). In WD, MSC-related studies have focused on ATP7B delivery, copper handling, cell resistance to copper toxicity, and modulation of hepatic inflammation or fibrosis, with little direct evidence for neurological recovery (331–333). These disorders should therefore not be pooled for efficacy claims. Their inclusion is intended to illustrate the potential adaptability of MSC/MSC-EV platforms whilst emphasising that mechanism, background standard of care, endpoint selection, and evidentiary threshold must remain disease specific.

To facilitate direct comparison across the heterogeneous clinical and product landscape, **Table 1** summarises representative completed and ongoing clinical programmes, and **Table 2** compares the principal MSC tissue sources and MSC-EV platform categories discussed above. These tables are intended as translational maps rather than evidence rankings; cross-trial efficacy comparisons remain inappropriate when manufacturing, dose, route, population, and endpoints differ.

**Table 1:** Representative clinical programmes of MSC- and MSC-EV-based therapy in neurodegenerative disorders.

Disease/programme	Product and source	Route and dose/regimen	Design/status	Primary endpoint and key finding	Ref./ID
AD: laromestrocel	Allogeneic bone-marrow MSCs	IV; 25×10^6 once or monthly ×4; 100×10^6 monthly ×4	Randomised, double-blind phase 2a; 49 treated; completed	Primary safety endpoint met; exploratory clinical, imaging and inflammatory signals require confirmation	(161)
AD: ahaMSC-Exos	Allogeneic adipose-MSC exosomes	Intranasal 2×10^8, 4×10^8 or 8×10^8 particles twice weekly for 12 weeks	Open-label phase I/II; n=9; completed	No treatment-related AEs reported; medium-dose cognitive signal; no significant amyloid/tau change	(88); NCT04388982
PD: allogeneic BM-MSC RCT	Allogeneic bone-marrow MSCs	IV 10×10^6 cells/kg; up to 3 infusions at 18-week intervals	Randomised, double-blind phase 2; n=45; completed	MDS-UPDRS-III responder analysis showed mixed dose-arm results; highlights need for batch potency assessment	(191)
ALS: MSC-NTF (NurOwn)	Autologous bone-marrow MSCs induced to secrete neurotrophic factors	Intrathecal at weeks 0, 8 and 16; nominal cell dose not reported in primary article	Randomised, double-blind phase 3; 196 randomised/189 treated; completed	Primary ALSFRS-R slope responder endpoint not met (32.6% vs 27.7%; P = 0.45); CSF biomarker target engagement observed	(124); NCT03280056
MSA-C randomised trial	Autologous bone-marrow MSCs	Combined intra-arterial/IV; 4×10^7 cells per injection	Randomised placebo-controlled; n=33; 360-day follow-up; completed	Smaller UMSARS worsening than placebo; intra-arterial administration produced small ischaemic MRI lesions	(301)
VaD pilot	Allogeneic human umbilical-cord MSCs	Three-course IV infusion; per-infusion dose not specified in abstract	Uncontrolled pilot; n=10; completed	Feasible; short-term MMSE/Barthel improvement was not sustained at 6 months; efficacy remains unproven	(316)
AD + anti-amyloid combination	Human MSCs added to standard anti-amyloid monoclonal antibody therapy	Single IV MSC infusion; registered dose not displayed in public summary	Pilot phase 2; recruiting at time of revision	Designed to test whether adjunct MSC therapy stabilises cognitive/functional decline; no efficacy results yet	NCT07512362

Representative rather than exhaustive. Trial-registry status was checked on 4 September 2026; registry-only programmes are listed for landscape context and are not treated as evidence of efficacy. AD, Alzheimer’s disease; ALS, amyotrophic lateral sclerosis; BM, bone marrow; IV, intravenous; MMSE, Mini-Mental State Examination; MSA-C, multiple system atrophy-cerebellar type; MSC, mesenchymal stromal cell; MSC-EV, MSC-derived extracellular vesicle; PD, Parkinson’s disease; UMSARS, Unified Multiple System Atrophy Rating Scale; VaD, vascular dementia.

**Table 2:** Comparative features of major MSC tissue sources and MSC-EV platform categories in neurodegenerative research.

Platform/source	Biological and practical features	Representative applications in this Review	Principal advantages	Key translational limitations
Bone marrow MSC/MSC-EV	Longest clinical history; adult source; potency can decline with donor age and expansion	AD, PD, ALS, MSA, VaD	Extensive manufacturing/clinical experience; established release frameworks	Invasive harvest; lower yield; age/disease imprinting; post-thaw and donor variability
Adipose MSC/MSC-EV	High tissue yield; common autologous source; EV platform already tested intranasally in AD	AD, PD, ALS; engineered EV studies	Abundant starting material; scalable EV production; practical autologous access	Metabolic comorbidity and donor age may alter potency; adipose heterogeneity; source/process-dependent cargo
Umbilical cord/Wharton’s jelly MSC/MSC-EV	Perinatal source with robust expansion and bankability	PD, ALS, VaD; broad preclinical neurodegeneration	Young-donor material; scalable allogeneic manufacturing; rapid availability	Donor/bank comparability; repeat-dose immunogenicity; source definitions and processing must be harmonised
Dental-tissue MSC/MSC-EV	Neural-crest-associated populations with neurotrophic potential; limited starting tissue	AD, PD, HD	Neurotrophic/neuronal-support phenotype in several experimental systems	Limited yield and donor availability; heterogeneous dental sources; few controlled neurological trials
Placenta-derived MSC/MSC-EV	Perinatal, potentially scalable allogeneic platform	Emerging neurological and vascular applications; limited direct neurodegenerative evidence	Non-invasive procurement; high expansion potential	Sparse disease-specific clinical evidence; placental region and isolation method influence phenotype
Olfactory-mucosa MSC/MSC-EV	Neural-crest/CNS-adjacent source; specialised trophic and immunomodulatory profile	PD and related parkinsonian models/programmes	Biological proximity to neural lineage; potential neurotrophic activity	Harvest and expansion standardisation; limited multicentre experience; source-specific potency not established
Engineered/preconditioned MSC-EV	Cargo loading, surface targeting, hypoxic/inflammatory/pharmacological priming or 3D culture	AD, PD, HD, ALS and targeted-delivery concepts	Potentially stronger potency, CNS targeting and reproducibility	Adds process complexity, off-target risk, stability/comparability requirements and regulatory burden

AD, Alzheimer’s disease; ALS, amyotrophic lateral sclerosis; HD, Huntington’s disease; MSA, multiple system atrophy; MSC, mesenchymal stromal cell; MSC-EV, MSC-derived extracellular vesicle; PD, Parkinson’s disease; VaD, vascular dementia.

## 5 Translational challenges

The principal barrier to MSC translation is not lack of biological activity but failure to convert variable biological activity into a reproducible medicinal product. The phrase “MSC therapy” currently encompasses products that differ in tissue source, donor health, isolation, culture medium, oxygen exposure, passage, harvest density, cryopreservation, thawing, viability, route, dose, and timing. Such variation can change the secretome, immunological behaviour, metabolism, and survival. Conventional identity markers do not reveal whether a batch possesses the function required for a neurological indication. Each programme therefore needs a target product profile and a potency matrix connecting critical quality attributes to a defined mechanism and clinical endpoint; a practical indication-linked matrix is provided in **Table 3**.

**Table 3:** Critical quality attributes (CQAs) linked to disease-relevant potency assays for neurological MSC/MSC-EV products.

CQA	Analytical/release assessment	Mechanism-linked potency assay	Neurological relevance	Development principle
Identity and tissue origin	Source traceability; phenotype panel; morphology; contamination testing	Not sufficient alone for potency	Prevents pooling biologically distinct MSC sources/products	Report source explicitly; identity must accompany functional testing
Viability and post-thaw fitness (cells)	Viable-cell count; apoptosis; recovery after thaw; aggregate size	Secretory response after recovery/licensing	Low viability can change effective dose, lung trapping and secretome	Dose should be based on viable, functionally recovered cells
Senescence/mitochondrial fitness	p16/p21, SA-β-gal or validated senescence panel; mitochondrial potential/respiration	Maintenance of trophic/immunoregulatory activity under stress	Especially relevant to aged/autologous AD, PD, ALS and VaD products	Define acceptable senescence burden linked to preserved function
Immunomodulatory potency	IDO/PGE2/TSG-6 or validated pathway markers after licensing	Suppression/reprogramming of activated microglia or inflammatory immune-cell assay	Inflammation-enriched AD/PD/ALS/MSA phenotypes	Assay should reproduce intended inflammatory context and predict *in-vivo* target engagement
Neurotrophic/neuronal protection	BDNF/GDNF/IGF-1 and secretome profile	Neuronal survival, neurite/axon maintenance or stress-rescue assay	PD, ALS, HD and early network-preservation strategies	Avoid single-factor release claims; use integrated functional rescue
Neurovascular/BBB potency	VEGF and vascular mediator profile; procoagulant screen	Endothelial survival, TEER/permeability, pericyte/tight-junction or angiogenic assay	VaD and vascular/inflammatory subtypes of AD/MSA	Demonstrate barrier/perfusion benefit without excessive angiogenic or thrombotic activity
EV identity, purity and cargo	Particle size/count; EV markers; non-vesicular protein/lipoprotein contaminants; cargo profile	Recipient-cell functional assay at defined particle/protein/source-cell-equivalent dose	All EV indications, especially intranasal/targeted programmes	Particle number alone is not potency; specify dose metric and functional unit
Haemocompatibility/procoagulant activity	Tissue factor; thrombin generation; complement/platelet interaction	Flow-based microvascular or validated haemocompatibility assay where relevant	High-dose IV/IA cell products and vulnerable cerebrovascular patients	Set route-specific release limits and monitor coagulation risk
Stability and comparability	Storage duration; freeze–thaw; container/transport; pre/post-change analytics	Side-by-side potency after storage or process change	Multicentre chronic dosing and commercial scale-up	A process change requires functional comparability, not identity markers alone

AD, Alzheimer’s disease; ALS, amyotrophic lateral sclerosis; BBB, blood–brain barrier; BDNF, brain-derived neurotrophic factor; CQA, critical quality attribute; GDNF, glial cell line-derived neurotrophic factor; IA, intra-arterial; IDO, indoleamine 2,3-dioxygenase; IGF-1, insulin-like growth factor-1; IV, intravenous; MSC, mesenchymal stromal cell; MSC-EV, MSC-derived extracellular vesicle; PD, Parkinson’s disease; PGE2, prostaglandin E2; TEER, transendothelial electrical resistance; TSG-6, tumour necrosis factor-stimulated gene-6; VaD, vascular dementia.

A target product profile should be established before confirmatory clinical development. It should specify the intended population, mechanism, route, dosing interval, critical quality attributes, release criteria, storage conditions, and clinically meaningful benefit. Without this discipline, manufacturing is often optimised for convenience and trial design proceeds before the active product has been defined. Comparability is equally important: a change in donor bank, medium, bioreactor, passage, cryoprotectant, thawing procedure, or EV purification may alter potency even when conventional identity tests remain unchanged. Analytical comparability should therefore include orthogonal functional assays and, when uncertainty remains, bridging pharmacology or clinical data (23, 33, 50, 58, 59, 64, 65).

CNS delivery presents a second structural problem. Intravenous administration is scalable and repeatable but exposes most cells to pulmonary retention and systemic clearance. Intra-arterial delivery may increase first-pass brain exposure but carries vascular risk. Intrathecal delivery bypasses the blood-brain barrier but produces uneven parenchymal distribution and procedural burden. Stereotactic injection offers local concentration at the cost of invasiveness and limited coverage. Intranasal delivery is attractive, particularly for EVs, but human pharmacokinetics are poorly defined. Route selection should be based on quantitative biodistribution, target anatomy, duration of action, repeat-dosing needs, and safety rather than convenience or precedent.

Dose-ranging should move beyond the assumption that more cells or particles produce greater efficacy. High cell concentrations can increase aggregation, complement activation, tissue-factor-mediated coagulation, and microvascular obstruction, whereas repeated low doses may better sustain a transient secretory effect. EV dose-response relationships can be non-linear because different cargo components engage distinct pathways and clearance mechanisms. Pharmacokinetic concepts for living cells and complex vesicles will not mirror those for small molecules, but exposure can still be characterised through labelled-product studies, circulating cargo, imaging, tissue sampling where feasible, and downstream pharmacodynamic markers. A rational schedule should be derived from the duration of biological effect rather than from arbitrary weekly or monthly conventions (33, 51–55, 199–204).

Disease and patient heterogeneity further dilute efficacy. Neurodegenerative diagnoses contain subgroups defined by genetics, protein pathology, vascular burden, immune state, disease stage, and progression speed. A product that modifies active inflammation may fail in a late stage dominated by irreversible neuronal loss, whilst a trophic product may have little effect when toxic protein production remains uncontrolled. Broad eligibility criteria increase statistical noise and obscure responsive phenotypes. Biomarker stratification should therefore be prospectively linked to mechanism, and interaction analyses should be pre-specified rather than performed after a negative trial.

Responder identification must be prospective. *Post hoc* subgroup findings after a neutral trial are vulnerable to multiplicity and regression to the mean, particularly when many biomarkers and clinical features are available. Candidate predictive markers should be selected from mechanism, validated analytically, and incorporated into stratification or enrichment before randomisation. The same principle applies to disease stage: early intervention is not automatically superior if the target pathway is inactive, and later intervention is not futile when a reversible inflammatory or vascular component remains. Precision development means selecting patients by active biology, not simply by diagnostic label or chronological stage (153, 157, 165, 205–208, 256, 266, 275–280).

Clinical endpoints frequently do not match the proposed mechanism. Short trials using subjective or slowly changing scales cannot determine whether a product modifies inflammation, neuronal injury, synaptic function, or vascular integrity. Conversely, biomarker changes without functional benefit may not be clinically meaningful. Trials should use layered endpoints: a proximal pharmacodynamic marker demonstrating target engagement, an intermediate marker of neural or vascular injury, and a patient-centred functional endpoint. Wearable sensors, digital measures, fluid biomarkers, molecular imaging, structural imaging, and neurophysiology can reduce noise, but they require validation and harmonised acquisition.

Statistical design must also reflect heterogeneous progression. Repeated-measures models, time-to-event outcomes, joint models of function and survival, and progression-slope analyses can be more informative than a single endpoint at an arbitrary visit. Missing data are rarely random in progressive neurological disease; dropout may reflect deterioration, caregiver burden, or adverse events. Intention-to-treat analysis, transparent estimands, sensitivity analyses for informative missingness, and pre-specified handling of rescue therapy are essential. Where sample sizes are constrained, as in rare disease, Bayesian or adaptive approaches may improve efficiency but cannot compensate for poorly characterised products or subjective endpoints (127, 256, 266, 275–280).

Reproducibility further requires complete separation of exploratory and confirmatory analyses. Multi-omic datasets can generate candidate responder signatures, but models derived from small trials are highly vulnerable to overfitting. Biomarker panels should be locked and validated in an independent cohort before they are used to claim precision treatment. Similarly, multiple doses, routes, time points, and clinical domains should not be searched *post hoc* for the most favourable result. Protocols and statistical analysis plans should be publicly accessible, and effect estimates with confidence intervals should be prioritised over isolated P values (5, 18, 127, 153, 157, 165, 256, 266, 275–280).

Safety assessment must reflect the specific product and route. Short-term infusion tolerance does not exclude delayed immune sensitisation, procoagulant activity, ectopic tissue responses, tumour-supporting effects, or risks from engineered cargo. Autologous products avoid some alloimmune concerns but can carry age- or disease-related dysfunction; allogeneic products improve scalability but require immunogenicity and repeat-dose assessment. EVs remove viable-cell risks but introduce uncertainties related to cargo heterogeneity, off-target distribution, and contaminants. Long-term surveillance and transparent reporting of neutral and adverse findings are necessary to counter publication and commercial bias. These interacting barriers are summarised in **Figure 4**.

**Figure 4:**
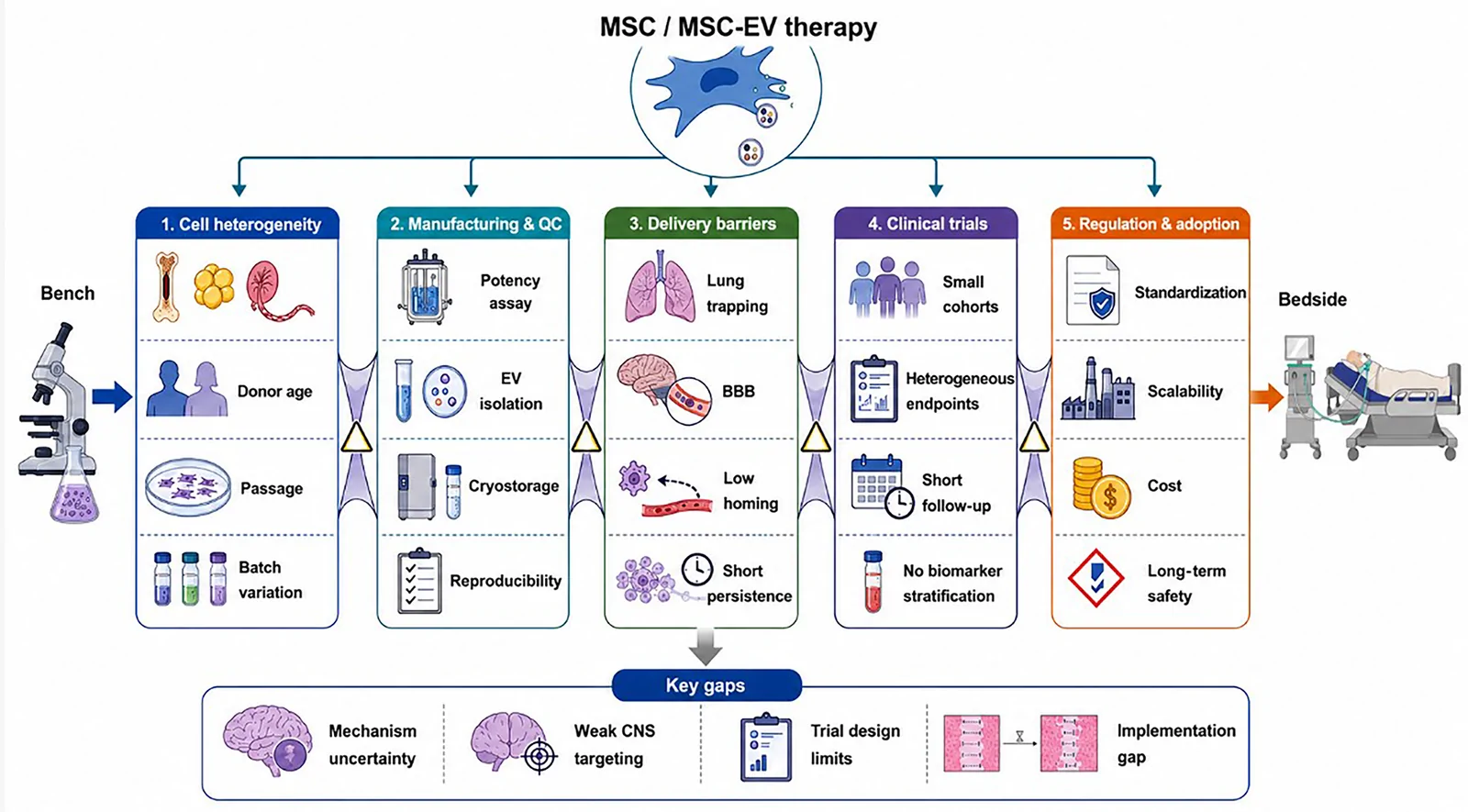
Translational barriers from bench to bedside in mesenchymal stromal cell (MSC) therapy. This schematic illustrates the major translational obstacles limiting the clinical application of mesenchymal stromal cells (MSCs) and MSC-derived extracellular vesicles (MSC-EVs) in neurodegenerative diseases. The developmental pipeline from bench research to bedside application is hindered by five key categories of barriers: (1) cell heterogeneity, including variability in tissue source, donor age, passage number, and batch-to-batch differences; (2) manufacturing and quality control limitations, such as inconsistent potency assays, challenges in extracellular vesicle (EV) isolation, cryostorage instability, and limited reproducibility; (3) delivery barriers, including pulmonary trapping, poor central nervous system (CNS) homing, blood–brain barrier (BBB) restriction, and short *in vivo* persistence; (4) clinical trial limitations, characterised by small sample sizes, heterogeneous outcome measures, short follow-up periods, and lack of biomarker-based stratification; and (5) regulatory and implementation challenges, including insufficient standardisation, limited scalability, high production cost, and unresolved long-term safety concerns. These multilevel barriers collectively contribute to the persistent efficacy gap between preclinical promise and clinical translation. A summary panel highlights key translational gaps, including mechanism uncertainty, weak CNS targeting, limitations in trial design, and implementation barriers. MSC, mesenchymal stem cell; MSC-EV, mesenchymal stromal cell-derived extracellular vesicle; CNS, central nervous system; BBB, blood–brain barrier; EV, extracellular vesicle.

Ethical and commercial governance are part of translational quality. Unregulated clinics commonly exploit the ambiguity of the term ‘stem cell’, cite preclinical publications as proof of benefit, and offer poorly characterised products without controlled follow-up. Academic overstatement can unintentionally support these practices. Manuscripts and consent documents should clearly distinguish established safety, preliminary biological activity, and unproven efficacy. Participants should be informed of product uncertainty, alternative care, long-term surveillance, and the possibility that manufacturing failure will prevent treatment. Conflict-of-interest disclosure and independent data monitoring are essential when investigators are also product developers (19, 56–60, 62–65, 69).

## 6 Precision regenerative strategies

### 6.1 Mechanism-defined product development

Future development should begin with a specific therapeutic hypothesis rather than the generic proposition that MSCs are regenerative. Investigators should define the dominant disease process to be modified, select a cell source or EV product with the appropriate functional profile, and establish assays that quantify that function. Multi-omic and single-cell analyses can identify active subpopulations and cargo signatures, but candidate markers must be reduced to practical release tests. Potency assays should be stable, rapid, scalable, and predictive of *in vivo* activity.Reference standards and cross-laboratory qualification are needed before multicentre trials.

Mechanism-defined development also requires deliberate deconvolution of the active product. For intact cells, investigators should compare viable cells with apoptotic cells, conditioned medium, EV-depleted secretome, purified EVs, and selected recombinant mediators where scientifically justified. For EV products, depletion or enrichment of candidate cargo and blockade of recipient pathways can identify necessary components. These experiments can reveal whether the therapeutic effect is distributed across a network or dominated by a tractable mediator. The result should inform both potency testing and product simplification: if a stable acellular fraction reproduces efficacy, retaining a complex living-cell product may be unnecessary; if host-responsive secretion is essential, EV substitution may sacrifice potency (31, 32, 34–43).

### 6.2 Engineering and preconditioning

Hypoxic, inflammatory, pharmacological, or three-dimensional preconditioning can enhance survival and secretory activity without permanent genetic alteration. Genetic engineering can increase neurotrophic factors, anti-inflammatory mediators, homing receptors, enzymes, or therapeutic RNA cargo. EVs can be loaded with nucleic acids or drugs and modified with targeting ligands. These strategies should not be adopted merely because they improve a single animal outcome. Enhancement must be linked to a measurable quality attribute, maintained after manufacturing and storage, and evaluated for off-target activity, genomic stability, tumour-supporting effects, and dose-dependent toxicity. Simpler modifications with reproducible benefit may be more translatable than highly complex constructs.

Preconditioning should be treated as a manufacturing operation, not an informal laboratory enhancement. Exposure intensity, duration, washout, harvest timing, and residual agents must be controlled because each can alter cell state and secretome composition. Genetic engineering requires additional safeguards for vector integration, transgene stability, inducibility, shedding, and off-target activity. Three-dimensional spheroids and bioreactors may increase yield and modify potency, but scale-up can change oxygen and nutrient gradients and produce a product that is not comparable with the laboratory prototype. Quality-by-design approaches should identify the process parameters most strongly linked to function and define an acceptable operating range before pivotal trials (163, 166–169, 172, 186, 209–215, 217, 218, 222–225).

EV standardisation requires its own quality strategy. Minimal characterisation should confirm vesicular identity and size distribution, quantify co-isolated proteins and lipoproteins, and assess sterility, endotoxin, residual nucleic acid, and process contaminants. Yet analytical purity alone is insufficient. A release assay should measure the intended biological function, and stability studies should determine whether freeze-thaw cycles, storage duration, container materials, or lyophilisation alter cargo and potency. Because particle counts can include inactive or contaminating structures, dose should be reported using multiple complementary metrics and related to source-cell production and functional activity (3, 4, 10, 12, 14, 36, 43, 216–225).

### 6.3 Targeted and repeatable delivery

Delivery platforms should be designed around anatomical distribution and treatment duration. For diffuse disorders, repeatable systemic or intranasal EV administration may be more practical than focal cell implantation. For local or compartmental disease, intrathecal or stereotactic approaches may be justified if exposure and benefit exceed procedural risk. Biomaterials, hydrogels, nanoparticles, magnetic guidance, and focused ultrasound may increase retention or barrier passage, but each adds manufacturing and regulatory complexity. Quantitative imaging or labelled-product studies should define where the product travels, how long it persists, and whether exposure correlates with pharmacodynamic response.

For chronic disease, repeatability may be more important than peak local exposure. A highly invasive administration that can be performed only once may be unsuitable when the product acts for days or weeks, whereas a lower-exposure route may be clinically useful if it is safe and repeatable. Patient burden, anaesthesia, device requirements, immunogenicity, and cumulative procedural risk should therefore be included in route selection. Targeted carriers and focused ultrasound may improve distribution, but they create combination products whose components must be evaluated jointly. Delivery technology should solve a demonstrated exposure problem rather than add complexity to compensate for an undefined mechanism (199–204).

The route question is inseparable from product format. Intact cells are large, deformable, and biologically responsive; EVs are nanoscale, heterogeneous, and subject to rapid uptake by the mononuclear phagocyte system. A delivery method optimised for one cannot be assumed to suit the other. Intrathecal cells may remain largely within cerebrospinal fluid spaces, whereas intrathecal vesicles may distribute more broadly but be cleared quickly. Intranasal cells face viability and epithelial-barrier constraints distinct from those of vesicles. Development programmes should therefore evaluate route-product combinations rather than rank routes in isolation and should report absolute target-tissue exposure where feasible (199–204, 216–225).

### 6.4 Biomarker-guided trials

Precision trials should enrich for patients in whom the proposed mechanism is active and potentially reversible. Candidate dimensions include genetic subtype, pathological protein confirmation, inflammatory or immune signature, neurofilament level, vascular burden, disease duration, and rate of progression. Adaptive or platform designs may compare products, doses, or combinations efficiently, but only if manufacturing is stable. Randomisation, blinding, allocation concealment, pre-specified analyses, and adequate follow-up remain indispensable. Natural-history data and external controls can support rare-disease development but should not replace concurrent controls when outcomes are variable or susceptible to expectation.

Trial architecture should allow early termination of biologically inactive programmes. A staged design can require predefined success at successive levels: acceptable product quality, demonstrable exposure, target engagement, evidence of reduced tissue injury, and finally clinical benefit. Failure to achieve a proximal pharmacodynamic endpoint should prevent escalation to a large efficacy trial. Conversely, a biomarker signal should not be used to bypass the need for patient-centred outcomes unless the biomarker is a validated surrogate. This discipline can reduce the recurrent pattern in which small safety studies with exploratory positive signals lead directly to underpowered efficacy trials (5, 18, 153, 157, 165, 191, 196, 205–208, 256, 266, 275–280, 295–304).

### 6.5 Rational combinations

MSCs are unlikely to correct every component of complex neurodegeneration. Combination therapy may pair their microenvironmental effects with interventions that directly target amyloid, tau, alpha-synuclein, mutant huntingtin, SMN deficiency, oxidative pathways, vascular risk, or rehabilitation-driven plasticity. The sequence and dose of each component should be mechanistically justified. A treatment that reduces inflammation might improve the function of subsequently administered cells; conversely, immunosuppressive drugs could blunt inflammatory licensing and reduce potency. Combination studies must therefore evaluate pharmacodynamic interaction and safety rather than assume additive benefit.

Combination development should begin with a mechanistic interaction map. For example, an anti-inflammatory drug may create a less hostile environment for transplanted cells but may also remove the inflammatory licensing signals required for MSC potency. Anti-amyloid or huntingtin-lowering therapy may reduce the primary insult and thereby extend the duration of trophic support, whereas simultaneous administration could change distribution or clearance. Rehabilitation and neuromodulation may amplify plasticity after biological protection, but their independent effects must be controlled. Factorial or sequential designs, supported by pharmacodynamic sampling, are preferable to empirically adding MSCs to an already complex regimen (158, 165, 209–225, 236, 238, 240–250).

### 6.6 Manufacturing and governance

A full-chain quality system should cover donor eligibility, tissue procurement, raw materials, isolation, expansion, passage limits, harvest criteria, cryopreservation, transport, thawing, final formulation, sterility, viability, identity, purity, potency, stability, and comparability after process change. For EVs, source-cell state, collection conditions, purification, particle characterisation, cargo, contaminant limits, and storage require equivalent control. Process changes should trigger analytical and, when necessary, clinical comparability studies because for living and complex biological products the process is inseparable from the product. Academic and commercial programmes should also register protocols, publish negative results, and clearly distinguish experimental treatment from proven care.

Regulatory convergence will be important because MSC and EV products are developed across jurisdictions with different definitions and release requirements. Internationally harmonised reporting of donor attributes, culture conditions, passage, viability, identity, potency, sterility, endotoxin, procoagulant activity, EV characterisation, dose, route, and concomitant treatment would immediately improve interpretability. Clinical-trial registries should include sufficient product detail to distinguish genuinely comparable interventions, and publications should identify the manufactured product rather than use 'MSC therapy' as though it were a single intervention. Shared reference materials, proficiency testing, and multicentre comparability exercises could further reduce laboratory-specific effects (23, 33, 50, 56–60, 62–65, 69).

The proposed transition is from empirically administered heterogeneous cells to precision products with defined composition, mechanism, potency, delivery, and responder population. This roadmap, including patient stratification, engineered-product optimisation, targeted delivery, standardised clinical trials, and rational combinations, is illustrated in **Figure 5**.

**Figure 5:**
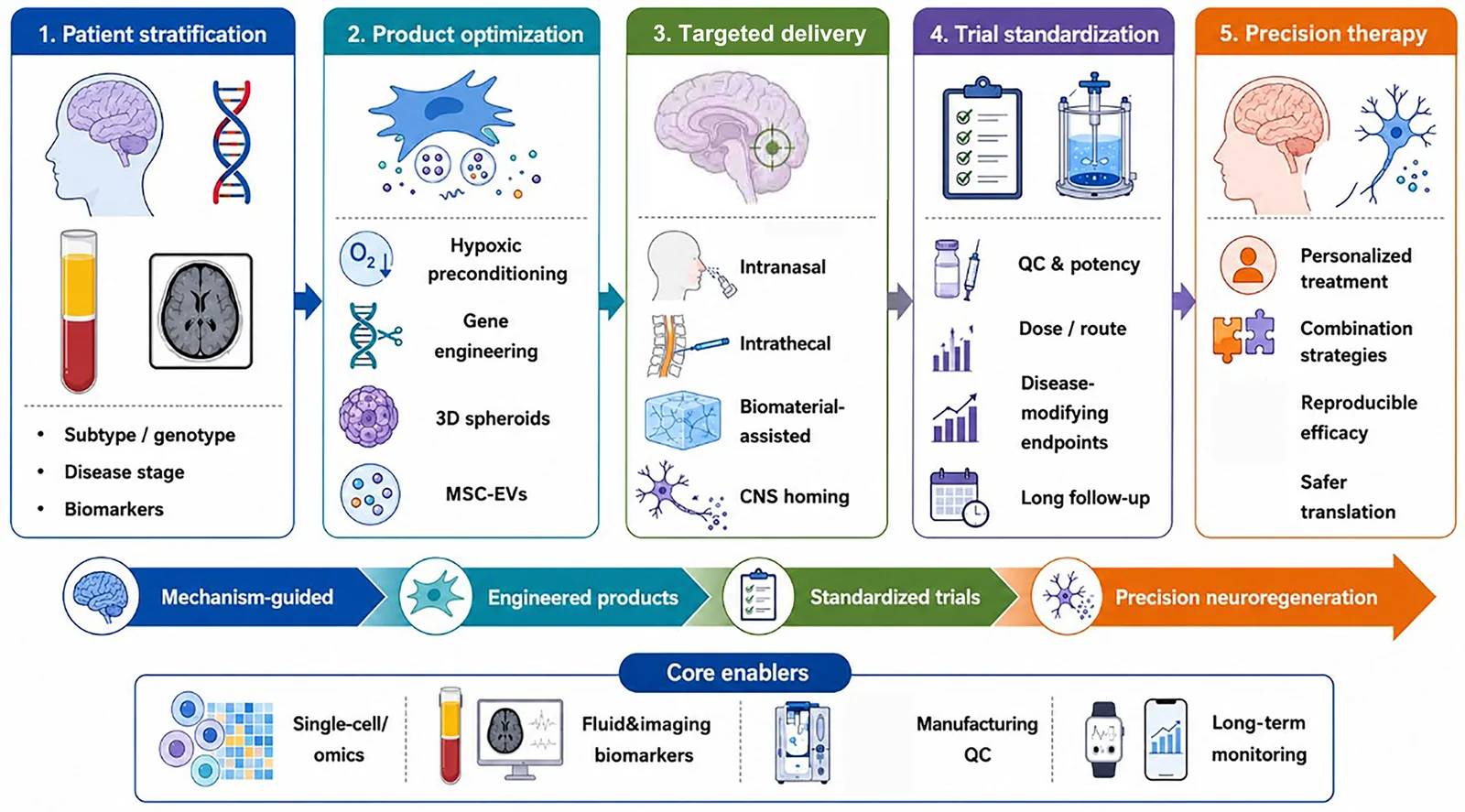
Next-generation precision MSC therapeutic roadmap. This schematic illustrates a next-generation precision medicine framework for mesenchymal stromal cell (MSC) and MSC-derived extracellular vesicle (MSC-EV) therapy in neurodegenerative diseases. The roadmap is organised into five sequential and interconnected stages: (1) patient stratification, incorporating disease subtype/genotype classification, disease stage assessment, and biomarker profiling to enable individualised therapeutic selection; (2) product optimisation, including hypoxic preconditioning, gene engineering, three-dimensional (3D) spheroid culture, and MSC-EV-based strategies to enhance therapeutic potency; (3) targeted delivery, featuring intranasal, intrathecal, biomaterial-assisted delivery approaches and improved central nervous system (CNS) homing efficiency; (4) trial standardisation, emphasising quality control (QC) and potency assessment, optimised dose and administration routes, disease-modifying endpoints, and long-term follow-up strategies; and (5) precision therapy, highlighting personalised treatment strategies, rational combination therapies, reproducible efficacy, and improved safety profiles. The bottom panel summarises key translational enablers, including single-cell and multi-omics technologies, fluid and imaging biomarkers, standardised manufacturing and quality control systems, and long-term clinical monitoring frameworks. Collectively, this roadmap outlines the transition from mechanism-guided research to engineered MSC products, standardised clinical evaluation, and ultimately precision neuroregenerative medicine. MSC, mesenchymal stem cell; MSC-EV, mesenchymal stromal cell-derived extracellular vesicle; CNS, central nervous system; EV, extracellular vesicle; QC, quality control; 3D, three-dimensional.

Clinical positioning should remain deliberately bounded. MSC-based products are most plausibly suited to biological states in which inflammation, trophic insufficiency, mitochondrial stress, or neurovascular dysfunction contributes materially to ongoing but potentially reversible injury. They are less likely to benefit disorders or stages dominated by completed neuronal loss, fixed network disconnection, or an uncorrected high-intensity genetic insult. This distinction provides a practical go/no-go rule for development and communication. It also protects patients from the misleading implication that a regenerative label guarantees anatomical restoration. Precision regenerative medicine should mean precise selection of a modifiable process, not expansion of therapeutic claims (3, 5, 17–20).

## 7 Conclusions and outlook

MSC-based therapy has progressed from a cell-replacement concept to a platform for transient, multimodal regulation of neuroimmune, metabolic, and neurovascular dysfunction. Preclinical studies across AD, PD, HD, ALS, MSA, and VaD provide substantial proof of biological activity, and early clinical studies generally support feasibility and short-term safety. Nevertheless, consistent disease-modifying efficacy has not been established. The main obstacles are heterogeneous products, uncertain dose and biodistribution, weak potency assays, biologically mixed trial populations, and endpoints that are poorly aligned with the proposed mechanism.

The field should therefore abandon universal claims and develop indication-specific products. Success will depend on functional potency assays, stable manufacturing, quantitative biodistribution, biomarker-guided enrolment, rigorous controlled trials, and transparent safety reporting. Engineered MSCs and EVs may improve targeting and reproducibility, whilst combination therapy may align microenvironmental regulation with causal disease interventions. These advances will be meaningful only when biological optimisation is matched by pharmaceutical discipline and clinically relevant evidence. Because for living cells and complex EV products the process is inseparable from the product, regulatory science should now prioritise shared reference materials, neurological indication-specific potency standards, inter-laboratory qualification/proficiency programmes, and formal comparability frameworks that remain valid across manufacturing sites and process changes.

The decisive question is not whether MSCs can produce favourable biological effects - they clearly can in many experimental systems - but whether a defined product can generate a predictable, clinically important effect in a specified patient population. Meeting that standard will probably narrow rather than broaden the field: some indications, products, and routes will be abandoned, whilst a smaller number of mechanism-aligned programmes may advance. Such contraction would represent scientific maturation rather than failure. It would replace class-level enthusiasm with product-level evidence and allow MSC-based therapies to be judged by the same standards of causality, manufacturing control, benefit, and risk applied to other advanced medicinal products.
